# Simultaneous distributed generation and electric vehicles hosting capacity enhancement through a synergetic hierarchical bi-level optimization approach based on demand response and Volt/VAR control

**DOI:** 10.1038/s41598-025-88635-8

**Published:** 2025-02-14

**Authors:** Zenhom M. Zenhom, Shady H. E. Abdel Aleem, Essam Aboul Zahab, Tarek A. Boghdady

**Affiliations:** 1 Electric Power Engineering Department, Ahram Canadian University, Giza, 12451 Egypt; 2 Department of Electrical Engineering, Institute of Aviation Engineering and Technology, Giza, 25152 Egypt; 3 Electric Power Engineering Department, Cairo University, Giza, Egypt; 4 Electric Engineering Department, Engineering and Information Technology College, Buraydah Private Colleges, Buraydah, 51418 Saudi Arabia

**Keywords:** Distributed generation hosting capacity, Electric vehicles hosting capacity, Demand response, Volt/VAR control, Bi-level optimization, And equilibrium optimizer, Energy science and technology, Engineering

## Abstract

In the context of sustainable development, electric vehicles (EVs) and renewable-based distributed generation (RDG) integration into the distribution networks (DNs) introduce various merits. They involve lowering harmful emissions, employing various types of energy sources, and encouraging the dependence of renewable energy. However, the two most challenging issues posing a grave danger to the technical operation of the DN would be the inappropriate integration of RDGs and EVs. Consequently, in order to guarantee safe operation, distribution system operator (DSO) is responsible for precisely identifying two crucial terms, distributed generation hosting capacity (DG-HC) and electric vehicles hosting capacity (EV-HC). Despite an increase in the amount of research on HC approaches, there is still a remarkable research gap in the discussion of models that effectively combine demand response (DR), smart inverters (SI) Volt/VAR control, DG-HC, and EV-HC targets simultaneously. This study offers a hierarchical bi-level optimization HC framework depends on both dynamic tariff-based DR and SI Volt/VAR control. In the lower layer, Participating customers’ load curves and EV aggregators’ charging demands are optimally adjusted, on a forecast basis, based on the proposed dynamic tariff. Nonetheless, the suggested multi-objective function—which includes DG-HC, EV-HC maximization, and loss minimization—is optimized by the DSO at the upper layer based on all of these optimal load curves. To further support the proposed objective function, DR is mixed with the optimal Volt/VAR controlling offered by SIs. In addition, the role of grid-connected EVs (GCEVs) on DG-HC increase is revealed with both uncoordinated and coordinated charging schemes. To check the proposed approach robustness, three types of loads are considered. Through comparison with three other optimization approaches, the effectiveness of the equilibrium optimizer (EO) is demonstrated when it is employed to solve the proposed optimization scheme. The suggested planning approach is applied on both the IEEE 33-bus test system, and a real DN with 59 buses in Cairo, Egypt. Several significant conclusions are validated by the obtained results. First, the DG-HC assessment differs significantly depending on whether the EV charging demand is considered or not. Considering the EV integration in IEEE 33-bus, the mean value of the optimal DG-HC increased by more than 133% during the day. Secondly, the implementation of the proposed dynamic-pricing DR program in the IEEE 33-bus DN significantly improved both the DG-HC and EV-HC, named as the combined DG-EV-HC, with improvements of around 34% and 27% for DG-HC and EV-HC respectively. Finally, in IEEE 33-bus, the combined DG-EV hosting capacity was improved by approximately 49.2% regarding DG-HC and 61.2% regarding EV-HC, using the proposed synergistic DR-Volt/VAR control enhancement technique.

## Introduction

### Background and motivation

Global warming, which is caused by greenhouse gas emissions, is a serious problem. By 2050, a number of nations, including the USA, EU, and Korea, have committed to achieving almost zero carbon emissions^1,2^. In fact, the largest portion of worldwide emissions in 2015 (42%) came from the production of electricity, and the transportation sector came in the second rank with 24% of global CO_2_ emissions^3^. In light of this, governments and citizens strongly supported distributed renewable energy sources and electric transportation as the cornerstones to break this bottleneck^4^. Thus, in recent years, there has been a notable increase in the deployment of both renewable-based distributed generation (RDG) and electric vehicles (EVs) into distribution networks (DNs)^5,6^. Massive RDG and EVs integration, however, presents a number of technical difficulties for the DNs. RDG expansion on a big scale may result in significant surplus generation and overvoltage issues. However, adding widespread EV charging may result in high demands and undervoltage issues^7^. Moreover, component overloading and a rise in system losses can result from both RDGs and EVs’ excessive penetration^8^. To ensure safe and dependable operation, the distribution system operator (DSO) is responsible for precisely defining the maximum permitted limit of RDG, known as DG-HC, and EVs, known as EV-HC^8^.

### Literature review

A mix of behavioural, policy, and technical measures is needed to achieve the challenging goal of increasing both DG-HC and EV-HC. For power systems to be resilient and sustainable—especially in light of the growing number of RDGs and EVs—these measures must be implemented synergistically^9^.

In the literature, SI Volt/VAR control has been employed to improve HC. In^10–13^ it has solely been applied to enhance DG-HC. While it has been combined with other enhancement methods in^14,15^. Lastly, to the best of our knowledge, it has not been directly employed for EV-HC enhancement research area yet. When it comes to^11^, authors employed the optimal Volt/VAR control of SIs using PSO to improve PV-HC in an Ecuadorian electric utility. Findings revealed that almost a 46% increase in PV-HC was achieved. Moreover, the effect of optimally adjusting the Volt/VAR function of SIs to tackle overvoltage issue and in turns enhance the PV penetration amount is examined in^10^. However, In^15^, SI Volt/VAR control was combined with time of use (TOU), one of the most popular DR programs, to tackle point of common coupling (PCC) issues caused by high PV penetration, such as overvoltage, increased losses, and voltage fluctuations. Mitigating these issues creates an opportunity to increase PV-HC. To the best of our knowledge, reference^15^ is the sole publication that combines DR and SI Volt/VAR control to enhance HC. In contrast, authors in^14^ suggested controlling smart inverters’ reactive power (Volt/VAR) optimally for both PV units and battery energy storage systems (BESSs) in order to enhance PV-HC. Different objectives are proposed concurrently, PV-HC maximization and voltage deviation minimization, therefore, the problem is defined as a multi-objective optimization. The slime Mold algorithm (SMA) is employed to tackle the optimization problem.

Even though the SI Volt/VAR control for DG-HC improvement has been presented throughout many publications, there are still important research gaps in this field. First, the suggested methods were solely considered from a residential loads’ perspective in most articles. Second, although SI Volt/VAR control assists in reducing overvoltage, which in turn presents a chance to increase DG penetration from PV or wind technologies, it was confined to improving PV-HC. Third, there has not been an obvious discussion in the literature yet regarding how SIs Volt/VAR control affects EV-HC enhancement. Lastly, DR programs have not been combined with SIs Volt/VAR control for HC enhancement yet. Despite the fact that SI Volt/VAR control was integrated with TOU, one of the DR programs, for PV-HC in^15^, this study had some shortcomings. The proposed approach depended on TOU, which is occasionally established months in advance and thus misses short-term changes in the supply-demand balance, was limited to PV only, and disregarded the integration impact of EVs.

On the other hand, Demand response (DR) is a critical tool that benefits customers, grid operators, and the environment by managing the consumption of energy, enhancing system reliability, and boosting the HC of RDGs and EVs^9^. Recently, DR programs have been employed as a low-cost, efficient alternative for enhancing HC rather than upgrading DN infrastructure, capacitor banks, and cable reinforcement^8^. When it comes to^16^, this work presented a technique for formulating a multi-objective optimization framework in which the DR is employed as an efficient tool to concurrently reduce energy losses and boost wind-HC. In^17^, a DR strategy based on load-shifting was recommended to increase the PV-HC. Using the suggested approach resulted in an average rise in PV-HC of almost 40%. In addition, DR is mixed with additional techniques to improve DG-HC, as presented in references^15,18,19^. The authors of^18^ provided an optimization technique that has various objectives, such as voltage imbalance, voltage profile enhancement, and DR cost minimization. A hybrid enhancement approach based on both DR and OLTC was coordinated to enhance the PV-HC. In comparison, both DR and capacitor banks are employed in^19^ to enhance wind-HC. The results show that using capacitor switching with DR increases the HC by roughly 27%. Although DR employment for DG-HC enhancement has been published in the literature, it is still in its early stages and has a number of research gaps. Most DR articles in the field of DG-HC enhancement relied solely on one form of DG technology—either wind or DG. Moreover, how various load types can be handled has not been discussed yet. Furthermore, there hasn’t been much explanation of the effectiveness of hybridizing DR with other methods like SI Volt/VAR control.

Unlike DG-HC, DR was employed in a number of publications for EV-HC improvement, as presented in^20,21^. In order to improve the DN reliability and raise the EV-HC, authors in^20^ suggested a DR program that relies on optimal coordination of EV charging and discharging operations. The authors presented various reliability indices as the limiting criteria of EV-HC, given the negative impact of excessive EV integration to DN on its reliability. The MC technique was introduced to determine the system’s reliability. However, a DR scheme relied on dynamic pricing was employed in^21^ to enhance EV-HC in a European DN.

Additionally, DG-HC and EV-HC have recently been the subject of numerous literature discussions; however, in the majority of these discussions, each is covered separately without an explanation of how each affects the evaluation of the other. There are only a few research^22–26^ focusing on how EV integration affects DG-HC assessment. In 2018, authors in^25^ first discussed the impact of aggregated GCEVs demands on the DG-HC of various technologies of DGs considering different load types. It was concluded that while the residential EVs load raises the DN’s peak demand, the DG-HC is not greatly impacted. Moreover, the DG technology determines how charging stations’ load affects the DG-HC. In the scenario when DGs are PV units, raising the load at the charging stations to its highest-level results in a 1.81% rise in DG-HC. However, if DGs are biomass and wind type, they can’t significantly boost the DG-HC of the DN. Although the previous study pioneered that research point, the authors did not consider any technique for enhancing either DG-HC or EV-HC. When it comes to^26^, authors employed a stochastic technique to determine PV-HC while guaranteeing that voltage constraints are fulfilled. The suggested approach integrates EVs to increase the PV-HC. The findings demonstrate that by allowing EV loads to reach 12% of peak loads at each PV location, the PV-HC in the IEEE 123-bus DN may be increased by about 4%. Authors in^24^ suggested a bi-stage optimization approach to optimally coordinate the operation of OLTC, the dispatch of EV aggregators, and the active and reactive DG power injections in order to calculate the maximum wind-HC. Findings indicate that, in comparison to an uncontrolled EV strategy, managing the power provided to EV aggregators can boost the DG-HC by almost 15%.

Furthermore, there are extremely few publications that simultaneously optimize EV-HC and DG-HC; only three are known to exist^5,9,27^. In 2020, authors in^5^ first proposed a strategy to assess the combined PV-EV hosting capacity. This study examines the impact of PV curtailment and EV charging coordination on both PV-HC and EV-HC. The EV-HC may be greatly increased by EV optimal charging coordination, while the hosting capacity for PV can be marginally increased. The PV-HC can be greatly increased by the PV curtailment, but not at all for EVs. Although the previous article pioneered that research point, the suggested method was limited to a single DG technology and was only seen from a residential view. To fill the previous gaps, authors in^27^ proposed a novel technique for the combined DG-EV hosting capacity assessment considering PV and wind DG technologies. Additionally, the results show that, in comparison to the scenario of EVs charging uncontrollably, a 9% rise in DG-HC can be achieved by regulating the power consumed by EV aggregators. It is important to note that the two aforementioned references enhanced the combined DG-EV hosting capacity by using the same hybrid enhancing strategy—EV charging coordination and active power curtailment. Nevertheless^9^, presents a novel synergistic enhancement strategy that combines DR and transmission expansion planning. The simulations also show that the maximum level of DG-HC is negatively impacted by the introduction of DR. Nonetheless, a noticeable and advantageous impact is noted in raising the minimum level of DG-HC throughout the day. However, an obvious rise in the EV-HC during peak hours is achieved by carefully choosing an adequate DR level. This means that there is a lack of research on the feasibility of combining various enhancement strategies to improve the combined hosting capacity of DG-EVs. In addition, authors in^28^ proposed a novel DR program which optimally dispatch RDG and charging and discharging of EVs to minimize the operating cost.

### Main contributions and paper organization

In light of the above and according to the authors’ best knowledge, simultaneous optimization of DG-HC, EV-HC, and system losses employing a synergetic enhancement technique based on both a bi-level dynamic-tariff DR approach and SI Volt/VAR control considering different load types haven’t been discussed so far. The work offered in this article involves the optimal placement and sizing of both RDG units, i.e., PV and wind technologies, optimal coordination of EV aggregated charging stations, optimal adjusting of load patterns, and optimal SIs Volt/VAR control. These are implemented simultaneously to maximize the DG-HC and EV-HC of the candidate DN and minimize total losses while keeping technical constraints within accepted bounds. Given the fact that overvoltage is considered the most concern that limits HC^8^, the suggested dynamic tariff approach has been specifically designed to mitigate the voltage rise problem. Therefore, in order to encourage customers to move their loads to the times that suffer from severe overvoltage and actively assist in fixing the issue, the DSO can modify the tariff to low values during overvoltage incidents. A bi-level optimization framework is proposed to implement the DR program. Through the lower-level layer, every customer locally modifies their load pattern in response to pricing functions and then sends the updated patterns back to the DSO. Based on the acquired optimal load curves, an optimized multi-objective function is created in the upper-level layer to concurrently maximize DG-HC and EV-HC and decrease total active power loss. Furthermore, optimal SIs Volt/VAR control is integrated to support the proposed multi-objective function further. The equilibrium optimizer (EO), a metaheuristic physics-based optimization method, is applied to tackle the optimization issue. Three types of loads are taken into consideration while assessing the robustness of the suggested technique: residential, commercial, and industrial. In comparison to some publications in the literature, Table 1 outlines the primary contributions of the current article. This is a summary of the main contributions provided by this paper:

- Assessment of the feasibility of combining SI optimal Volt/VAR control with the dynamic tariff DR program to improve the simultaneous DG-HC and EV-HC, named as the combined DG-EV-HC.
- Evaluation of the suggested technique’s robustness while considering various load types, such as commercial, industrial, and residential.
- The suggested framework is implemented using a hierarchical optimization technique that breaks down the planning model into multiple levels to simplify the problem and produce excellent results.
- Apply the EO to handle the optimization problem and compare it with four alternative approaches.
- Assess the scalability of the suggested method by testing it on a real DN with 59 buses in Cairo and the IEEE 33-bus test system.

The paper’s next sections are arranged as follows. The dynamic pricing formulation, the suggested optimization framework for the DR program, the selected DG technologies modeling, and the Volt/VAR optimum control are all explained in depth in Sect. 2. A summary of the EO optimizer is provided in Sect. 3. Test systems employed, cases examined, outcomes of simulations, discussions, and an assessment of the optimization algorithm’s performance are covered in Sect. 4. Section 5 provides the article’s conclusions and presents some future work.

**Table 1:** Summary of the primary contributions provided by the present work and some publications in the literature.

References	Year	Enhancement techniques	Solved strategy	Research gaps
^14^	2021	PV and BESS SI’ optimal Volt/VAR control was applied to enhance PV-HC	The multi-objective optimization solved by SMA	Disregarding EVs charging demand effect. Considering only one DG technology (PV).
^15^	2021	Mixing SI Volt/VAR control with TOU tariff to mitigate high PV penetration issues and, in turn, enhances PV-HC	Quasi-static time-series simulations carried out with the OpenDSS software.	Limited to the PV technology. EVs demand effect was ignored. The selected DR program, TOU, has some limitations.
^17^	2022	A load-shifting-based DR program is applied to enhance PV-HC	A suggested optimization framework based on overvoltage mitigation by giving subsidies.	Restricted to residential loads. Scalability was not verified (IEEE 15-bus was the sole test system). Overvoltage issue was the sole concern (disregarding other technical concerns)
^29^	2024	The consideration of background voltage improves the accuracy of HC assessment	The impact of two novel aspects, the background voltage and high impedance nodes is considered in PV-HC calculation.	Limited to the PV technology. Disregarding EV charging load in the proposed study.
^30^	2024	EV aggregators’ optimal charging and discharging coordination	A novel cooperative vehicle-to-vehicle sharing method is presented to optimize the utilization of EVs’ charging and discharging capacities in conjunction with PV-based DGs.	Limited to the PV technology. Restricted to residential loads.
^31^	2024	Novel three hybridization schemes were suggested to find better EV-HC values.	A proposed model seeks to maximize the EV-HC, reduce greenhouse gas emissions, and optimize net present value via a novel hybrid optimization method based on both the Marine Predators Algorithm and the Honey Badger Algorithm.	Limited to the PV technology. Disregarding variation of load types.
^32^	2024	EV aggregators’ optimal charging and discharging coordination via V2G technology	A novel approach for PV-EV/HC assessment is proposed, considering V2G.	Limited to the PV technology. No enhancement technique is applied to improve the assessed HC.
^21^	2022	A dynamic-tariff-based DR approach was proposed to improve EV-HC	A hierarchical bi-level optimization solved by the evolutionary algorithm	Limited to home charging, disregarding charging stations. Limited to residential load type.
^24^	2020	EV aggregators’ optimal charging coordination for Wind-HC enhancement	The problem was modeled as a bi-level mixed-integer linear programing.	Limited to wind DG technology. Disregarding EV-HC term in the objective function.
^5^	2021	A mix of PV curtailment and EV aggregators’ optimal charging coordination to enhance the combined PV-EV/HC	Applying a novel graphical approach, the lowest and highest voltages as functions of PV and EV penetration were formulated using linear regression.	Viewed from the domestic level only. The results’ poor quality due to their reliance on linear regression contains errors at several points. Limited to PV technology.
^27^	2021	Combining PV curtailment with the optimal charging coordination of EV aggregators to improve the combined DG-EV-HC	An approach relying on the greedy randomized adaptive search and tabu search (GRASP-TS) algorithms.	Disregarding variation of load types. Disregarding residential EVs charging demand.
Current work		A synergistic approach for enhancing the combined DG-EV-HC by mixing Volt/VAR control with a dynamic tariff-based DR program	A hierarchical bi-level optimization solved by EO	-

## Methodology

**Fig. 1:**
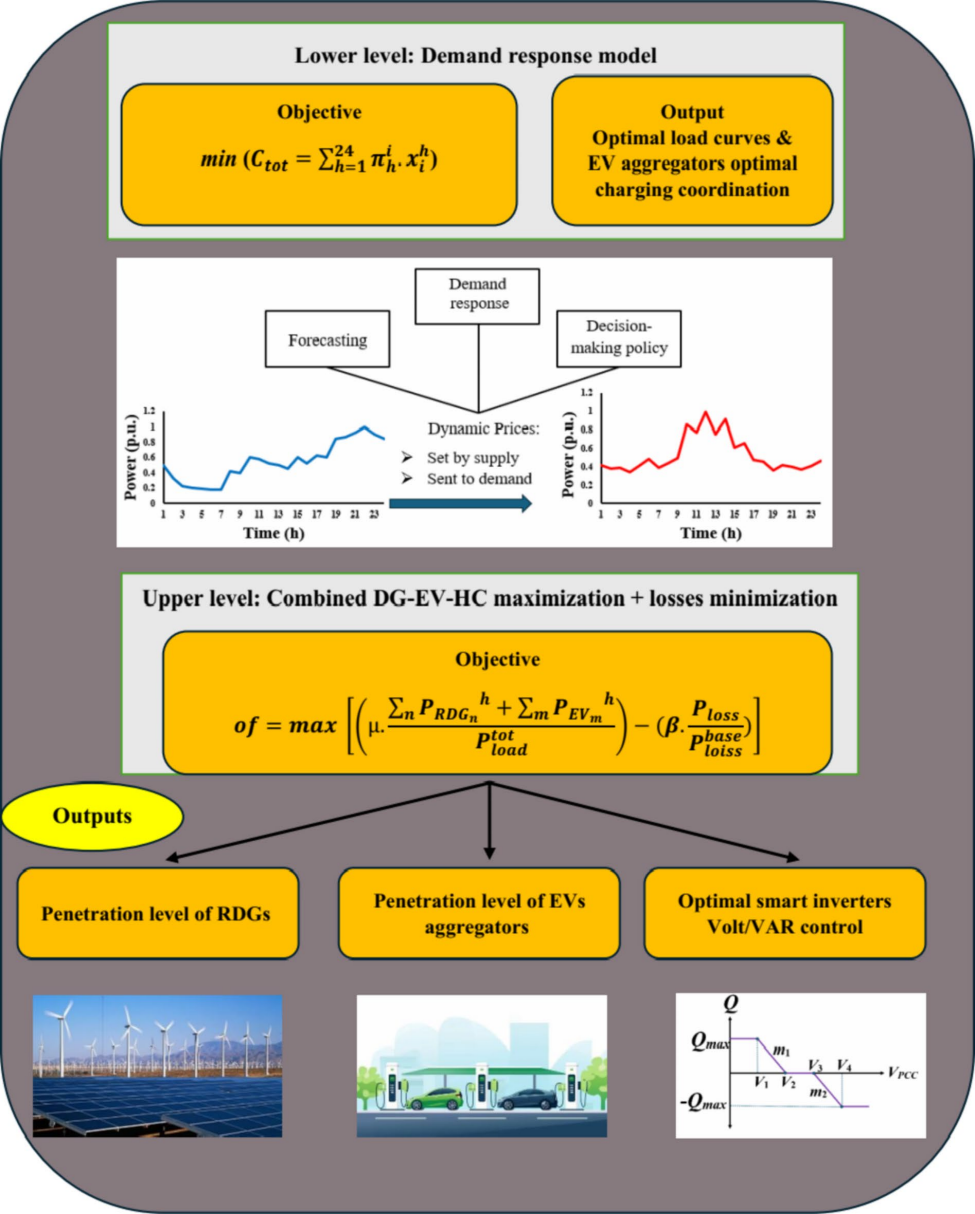
Graphical representation of the proposed framework for mixing the combined DG-EV-HC, DR, and SI Volt/VAR control.

The suggested framework for combining HC, Volt/VAR control of SIs, and DR is shown graphically in Fig. 1. The proposed approach presents a bi-level hierarchical optimization model that relies on SI Volt/VAR management and dynamic tariff-based DR. The lower level optimizes, on a forecast basis, the load curves of participating customers and the charging demands of EV aggregators using the suggested dynamic tariff. However, the DSO at the upper layer optimizes the proposed multi-objective function based on all these optimal load curves, which include DG-HC, EV-HC maximization, and loss minimization. In order to reinforce the suggested objective function, DR is combined with SIs’ ideal Volt/VAR regulating.

### DG units modelling

Regarding wind-powered DG, the output power and wind speed are directly related. Equation (1) ^27,33^ can be employed to calculate the produced active power ($\:{P}_{WT}$) from the wind turbine to be a function of wind speed (*V*) according to wind speed data gathered at various intervals^27^, where $\:{V}_{cut-in}$, $\:{V}_{rated}$, and $\:{V}_{cut-out}$ are the turbine’s cut-in, rated, and cut-out wind speeds, accordingly. Furthermore, $\:{P}_{rated}^{WT}$ represents the wind turbine unit’s rated power.

1 $${P_{WT}}=\left\{ {\begin{array}{*{20}{c}} 0&{V<{V_{cut - in}}} \\ {P_{{rated}}^{{WT}}\left( {\frac{{V - {V_{cut - in}}}}{{{V_{rated}} - {V_{cut - in}}}}} \right)}&{{V_{cut - in}} \leqslant V \leqslant {V_{rated}}} \\ {P_{{rated}}^{{WT}}}&{{V_{rated}} \leqslant V \leqslant {V_{cut - out}}} \\ 0&{V>{V_{cut - out}}} \end{array}} \right.$$

Regarding PV-powered DG, solar radiation is the only factor influencing the PV unit’s output power. Equation (2) ^27,33^ can be used to calculate the produced active power ($\:{P}_{PV}$) being a function of solar radiation (*G*), where $\:{G}_{std}$, $\:{G}_{c}$ represent the solar radiation at standard circumstances and specified radiation levels, respectively.

2 $$\:{P}_{PV}=\left\{\begin{array}{cc}{P}_{rated}^{PV}\left(\frac{{G}^{2}}{{G}_{std}{G}_{c}}\right)&\:0<G<{G}_{c}\\\:{P}_{rated}^{PV}\left(\frac{G}{{G}_{std}}\right)&\:G\ge\:{G}_{c}\end{array}\right.$$

### Proposed dynamic-pricing function

This paper’s main contribution is implementing a dynamic tariff scheme designed to improve voltage-restricted HC by reducing voltage rise problems. First, load flow equations are applied using the day-ahead solar irradiance and wind speed data. This makes it possible to determine bus voltages without taking the HC restrictions into account. The maximum voltage at each hour *h* is then found and is represented by the symbol ($\:{V}_{h}^{max}$). It is noted that this maximum voltage exceeds the upper permissible boundary( $\:{V}_{UB}$) for most of the hours. Next, using Eq. (3), compare the highest recorded bus voltage with the higher allowed limit to get the percentage of voltage rise ($\:{V}_{rise}^{h}$) at hour *h*.

3 $$\:{V}_{rise}^{h}=\frac{{V}_{h}^{max}-{V}_{UB}}{{V}_{UB}}$$

An exponentially inverse relationship is developed between the dynamic tariff and the percentage of voltage rise in order to compute the suggested hourly dynamic tariff for a customer *i* ($\:{\pi\:}_{h}^{i}$). The dynamic tariff that is proportionate to the amount of voltage rise can be determined using this relationship, as shown in Eq. (4), where $\:{\stackrel{-}{\pi\:}}_{i}$ is the mean of the previous tariff based on the *ith* load.

4 $$\:{\pi\:}_{h}^{i}={e}^{-{V}_{rise}^{h}}\cdot{\stackrel{-}{\pi\:}}_{i}$$

### Optimization model proposed for DR

A multi-objective hierarchical two-level time-varying optimization formulation is developed at the upper-level stage, also called the master phase, with the aim of maximizing the combined DG-EV-HC and minimizing overall active power loss. The goal of the lower-level stage, also known as the slave phase, is to minimize each customer’s daily electricity consumption costs. The DG units’ total installed capacities divided by the total load demand equals the hourly (*DG-HC*). Equation (5) can be employed to explain this, where the hourly value of (*DG-HC*) represented by ($\:{DG-HC}_{index}^{h}$). When it comes to Eq. (6), the hourly value of (*EV-HC*) represented by ($\:{EV-HC}_{index}^{h}$) can be computed by dividing the total EVs aggregators charging demands for both charging stations, denoted by $\:{EV}_{c}$, and residential EVs aggregators, denoted by $\:{EV}_{r}$, connected for all buses over the total load demand. The total number of buses denoted by *q*.

5 $$\:{DG-HC}_{index}^{h}=\left(\sum\:_{n}{{P}_{{WT}_{k}}}^{h}+\sum\:_{m}{{P}_{{PV}_{i}}}^{h}\right)/{P}_{load}^{tot}$$

6 $$\:{EV-HC}_{index}^{h}=\left(\sum\:_{q}{EV}_{r}+\sum\:_{q}{EV}_{c}\right)/{P}_{load}^{tot}$$

where the produced active power from the *kth* WT at hour *h* is represented by ($\:{{P}_{{WT}_{k}}}^{h}$), and the produced active power from the *i*th PV unit at hour h is represented by ($\:{{P}_{{PV}_{i}}}^{h}$). Furthermore, the hourly power loss index, denoted as ($\:{Ploss}_{index}^{h}$), can be computed by dividing the total active power loss at hour *h* ($\:{Ploss}^{h}$) by the total active power loss at the base case ($\:{Ploss}_{base}$), using the formula provided by Eq. (7).

7 $$\:{Ploss}_{index}^{h}=\frac{{Ploss}^{h}}{{Ploss}_{base}}$$

This multi-objective bi-level optimization model aims to minimize the cost of each customer’s electricity consumption at the lower level while concurrently determining the best values for DG-HC, EV-HC, and active power loss at the higher level. This offers the opportunity to operate the system more affordably, reduce power losses, as well as optimal utilization of DG resources.

#### Upper-level optimization model

At this level, as indicated by Eqs. (8)–(11), a multi-objective function is created to concurrently maximize the DG-HC, EV-HC, and reduce the overall active power loss.

8 $$\:{Of}_{1}:\text{max}\left({DG-HC}_{index}^{h}\right)$$

9 $$\:{Of}_{2}:\text{max}\left({EV-HC}_{index}^{h}\right)$$

10 $$\:{Of}_{3}:min\left({Ploss}_{index}^{h}\right)$$

11 $$\:Of={w}_{1}{Of}_{1}+{w}_{2}{Of}_{2}+{w}_{3}{Of}_{3}$$

subject to:

12 $$\:{P}_{WT}\le\:{P}_{WT}^{max}$$

13 $$\:{P}_{PV}\le\:{P}_{PV}^{max}$$

14 $$\:{V}_{min}<\left|{V}_{i}^{h}\right|<{V}_{max},\:\forall\:\:i,h\:$$

15 $$\:\left|{I}_{i}^{h}\right|<{I}_{max},\:\forall\:\:i,h\:$$

16 $$\:{Ploss}^{h}<{Ploss}_{base}$$

17 $$\:\sum\:_{i=1}^{n}{w}_{i}=1$$

where $\:{P}_{WT}^{max}$,$\:{\:P}_{PV}^{max}$ are the upper limit of the wind-based and PV-based RDG units, respectively. Moreover, $\:\left|{V}_{i}^{h}\right|$ is the voltage magnitude of bus *i* at *h*, while $\:\left|{I}_{i}^{h}\right|$ is its current magnitude.

The constraints of the upper-level optimization model are described by means of a series of equations, notably (12) to (16). The rated values, denoted by Eqs. (12) and (13) of the DG units set a limit on their output power to guarantee safe and effective operation. An upper and lower allowable limits are set in order to control the magnitude of bus voltages during the course of the day, as stated in Eq. (14). Moreover, Eq. (15) limits the maximum current that can pass through a feeder in a given hour so as not to surpass the ampacity of the cable. Additionally, Eq. (16) places a limit on the total active power loss, preventing it from going above the value in the basic scenario. Together, these limitations restrict the system’s behaviour and performance, ensuring dependable and controlled functioning. Finally, the proper selection of weighting factors in multi-objective functions is essential to precisely validate objectives’ prioritizing, control objectives’ trade-off, and identify the most influential objectives. As seen in Eq. (17), the sum of all weights is assumed to equal 1 for simplicity and confirmation of normalization.

#### Lower-level optimization model

The objective of this phase is to reduce the typical daily cost of consuming electricity and EVs aggregators charging cost. The dynamic pricing offered by the DSO is taken into account by this objective function. Relied on the optimal load curves and charging coordination that resulted from this optimization model, the clients and EVs aggregators can modify and redistribute their load profiles. Consequently, the following equations can be used to define the lower-level optimization problem:

18 $$\:\text{min}C=\sum\:_{j=1}^{q}\sum\:_{h=1}^{24}{\pi\:}_{h}^{j} \cdot {x}_{j}^{h}$$

subject to:

19 $$\:\sum\:_{h=1}^{24}{x}_{j}^{h}=\sum\:_{h=1}^{24}{l}_{j}^{h}\:,\:\:\:\forall\:\:j,h\:\:$$

20 $$\:{{l}_{{j}_{min}}\le\:x}_{j}^{h}\le\:{l}_{{j}_{max}},\forall\:\:j,h$$

while *C* represents the total daily cost of electricity consumption, and $\:{\pi\:}_{h}^{j}$, which varies depending on the kind of load (residential, commercial, industrial, or EV aggregator), represents the dynamic tariff of load bus *j* at hour *h*. Furthermore, $\:{x}_{j}^{h}$ hand $\:{l}_{j}^{h}$ indicate the original and modified loads for customer *j* at hour *h*, respectively. The original loads’ minimum and maximum values are shown by the variables $\:{l}_{{j}_{min}}$, $\:{l}_{{j}_{max}}$, respectively. As may be observed, additional constraints are introduced by the lower-level optimization problem, as stated in Eqs. (19) and (20). Equation (19) guarantees the consistency of the total adjusted loads with the total original loads for every day. This restriction ensures that any changes made to the loads won’t cause the distribution of the loads to change overall from the original load distribution. However, Eq. (20) places a limit on the modified load for the day. This restriction has two functions. Firstly, it makes sure that the modified load never falls below the initial load’s minimum, preserving economic dispatch. Secondly, it guarantees that the current utilized during peak hours does not exceed the planned feeder ampacity by preventing the adjusted load from surpassing the maximum limit of the original load.

### Smart inverter modeling

**Fig. 2:**
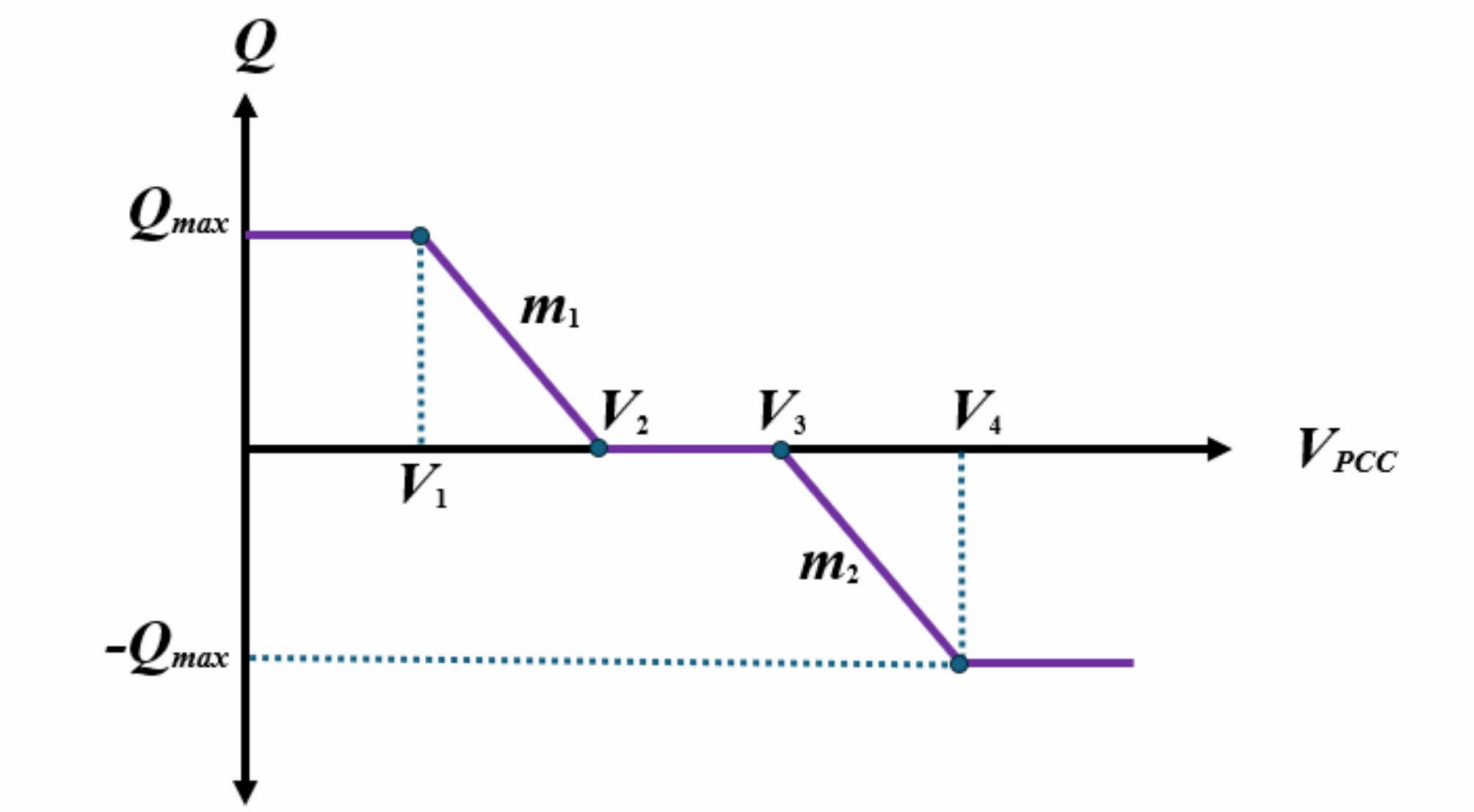
SI Volt/VAR control setting curve.

Converting DC power to AC power is a common application for traditional PV inverters, but a growing majority are expecting the SIs to have additional functions^14^. Overvoltage is a major concern when integrating solar PV power into the DN^34^. Several settings, including Volt/Var control, Volt/Watt control, Frequency/Watt control, and fixed power factor setting, are available on SIs to deal with voltage regulation. Volt/Var control is regarded as the most utilized feature among these settings^34,35^. A graphic illustration of the Volt/Var control is shown in Fig. 2. The SI modifies the reactive power injection or absorption in accordance with the bus voltage levels to guarantee appropriate regulation^34^. The minimum and maximum acceptable voltage limits are denoted by the variables V1 and V4, respectively. If the point of common coupling (PCC) voltage is less than or equal to V1, the SI will raise the voltage by adding the most reactive power possible to the grid. When the PCC voltage is between *V*_1_ and *V*_2_, the quantity of reactive power injected by the SI follows the slope of the line (*m*_1_). A dead-band zone is formed when the PCC voltage is between *V*_2_ and *V*_3_, meaning that the SI is not required to do anything more. The SI will absorb reactive power from the DN in accordance with the slope line (*m*_2_) in order to lower the DN voltage if the PCC voltage is between *V*_3_ and *V*_4_. The SI will absorb a maximum of the reactive power from the DN when the PCC voltage approaches or surpasses *V*_4_^14,34,35^. The optimization procedure in this study focuses on identifying the optimum control setting curve points (*V*_2_, *V*_3_) by adjusting the two slopes, *m*_1_ and *m*_2_, as stated in (21) and (22).

21 $$\:{V}_{2}={V}_{1}-\frac{{Q}_{max}}{{m}_{1}}$$

22 $$\:{V}_{3}={V}_{4}+\frac{{Q}_{max}}{{m}_{2}}$$

## Equilibrium optimization algorithm

The dynamic balance of mass in a control volume serves as the basis for the optimization method known as the equilibrium optimizer (EO), whose particular optimization procedure is as follows^36^:

### Initialization

Like the majority of meta-heuristics, EO initiates the optimization process with the initial population. Equation (23) illustrates how the starting position is uniformly and randomly built in the search space based on the quantity and dimension of particles.

23 $$\:{c}_{n}={c}_{min}+{rand}_{n}\left({c}_{max}-{c}_{min}\right),\:\:\:\:\:\:n=\text{1,2},\dots\:,m\:\:\:\:\:$$

where $\:{c}_{min}$, and $\:{c}_{min}\:$are the lower and upper boundaries respectively, and $\:{rand}_{n}\:$represents a random vector ranging from [0,1], and *m* is the population number.

### Equilibrium pool and candidates

As indicated by Eq. (24), the balancing pool gathers the highest four particles in the fitness value of the present solution. It employs their mean value as the candidate solution for the updated solution.

24 $$\:{c}_{eq}^{pool}=\left\{{c}_{eq}^{1},{c}_{eq}^{2},{c}_{eq}^{3},{c}_{eq}^{4},{c}_{eq}^{av}\right\}$$

while $\:{c}_{eq}^{1},{c}_{eq}^{2},{c}_{eq}^{3},{c}_{eq}^{4}$ represent the best solutions found in the present iteration, and $\:{c}_{eq}^{av}$ represents their mean value.

### Exponential factor (F)

The exponential factor, whose precise calculation formula is illustrated in Eqs. (25)–(27), could effectively balance the capacities for local development and global exploration^36^.

25 $$\:F={e}^{-\lambda\:(t-{t}_{o})}$$

26 $$\:{t}_{o}=\frac{1}{\lambda\:}\text{ln}\left[-{k}_{1}sign\left(rand-0.5\right) \cdot \left(1-{e}^{-\lambda\:t}\right)\right]+t$$

27 $$\:t={\left[1-\frac{l}{{l}_{max}}\right]}^{\left({k}_{2}\frac{l}{{l}_{max}}\right)}$$

where the weighting factors for global exploration are, respectively, $\:{k}_{1}$ and $\:{k}_{2}$. In general, their values are 2 and 1, respectively; λ is a random number ranging between [0, 1], $\:l$, and $\:{l}_{max}$ denote the present and maximum iterations, respectively.

### Generation rate (G)

One crucial factor in the mass balance equation is the generation rate. It is developed to further enhance the local development solution, as illustrated in Eq. (28) ^36^.

28 $$\:G={G}_{o}{e}^{-\beta\:(t-{t}_{o})}$$

where $\:{G}_{o}$ is the initial condition and $\:\beta\:$ represents a decay parameter.

### Update formula

**Fig. 3:**
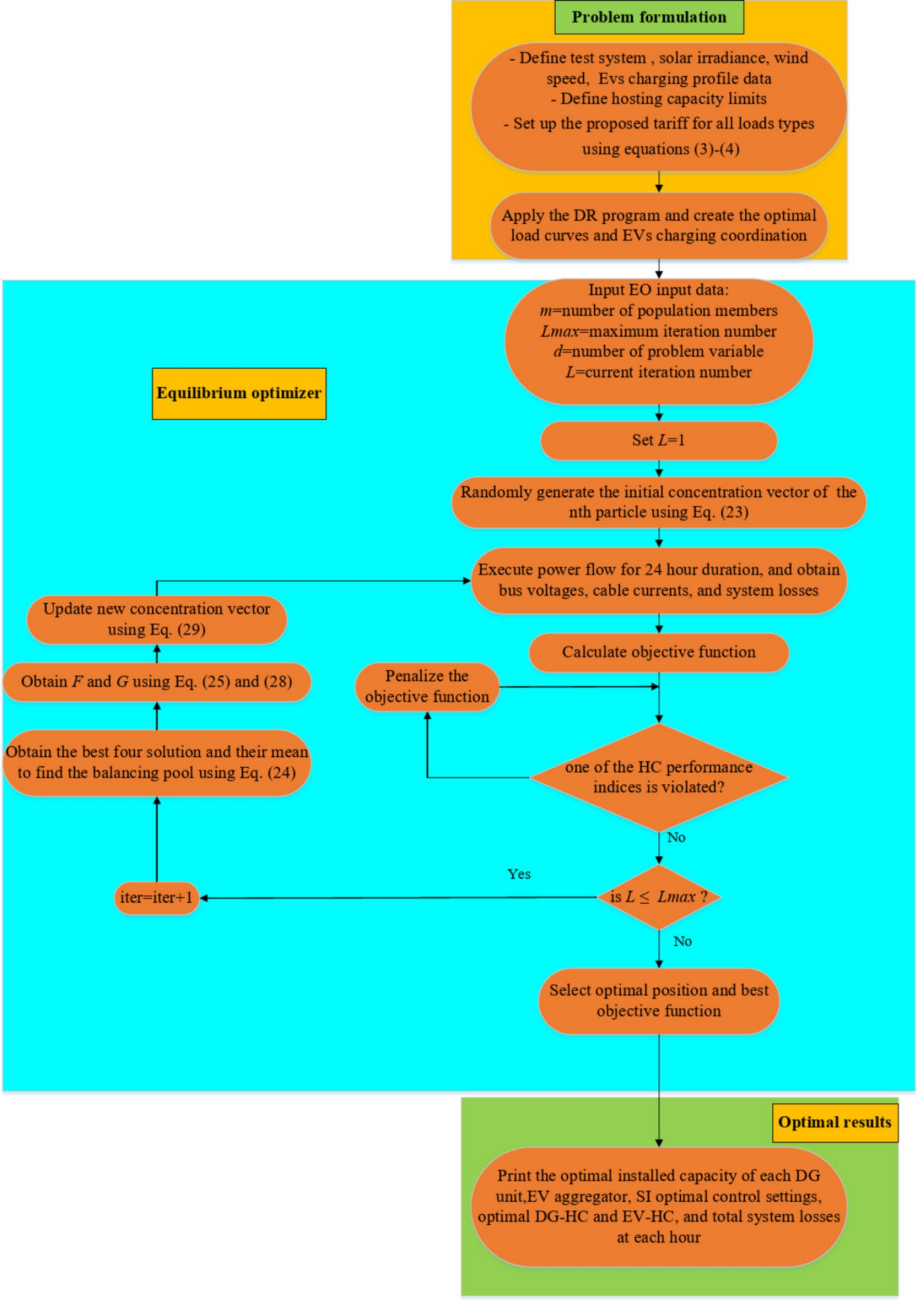
Flowchart of the EO method.

Equation (29) defines the modified EO formula after the original physical theory has been improved and abstracted^36^.

29 $$\:C={C}_{eq}+F\left(C-{C}_{eq}\right)+\frac{G}{\lambda\:V}(1-\text{F})$$

where a random selection of $\:{C}_{eq}$ occurs from the equilibrium pool and $\:V$ is set to be a unit. A flowchart describing the suggested methodology is shown in Fig. 3. The procedure starts with gathering pertinent data, such as load, solar irradiation, EV charging loads, and power system data. Then, the EO population individuals, the number of optimization variables, and the maximum iterations number are supplied to the optimization technique, and the random beginning placements of the particles are generated. The EO particles correlate to the problem variables to be optimized, such as the DG units installed capacities, EVs aggregators charging demand, and the Volt/VAR control settings of SIs. Then, the power flow equations are carried out for 24 h, ensuring that the limitations for each time interval stay within the acceptable range. The objective function is penalized if the limiting factors are violated. After that, the particles’ positions are updated repeatedly until the maximum number of iterations is fulfilled. Ultimately, the particle placements that produce the best objective function are chosen.

## Case description and results discussion

To show the effectiveness of the proposed synergetic technique, MATLAB R2021a software was used to execute several simulations on a laptop computer with an Intel Core i7 processor running at 3 GHz and 8 GB of installed RAM. The simulations covered different test systems and case studies, and a detailed analysis and discussion of the numerical results were conducted. Furthermore, by contrasting with other optimization methods, the effectiveness of the employed optimizer, EO, was assessed. The test systems, case studies considered, numerical results, and discussions performed in comparison to other optimization methods are covered in more detail in the ensuing subsections.

### Test systems

The IEEE 33-bus DN^37^ was employed to assess the suggested method’s effectiveness. This system has a base apparent power of 10 MVA and a base voltage of 12.66 kV. Its maximum overall load is 2.30 MVAR and 3.715 MW. In the base-case scenario, the system losses are about 136 kW in total when neither DGs nor EVs were integrated.

**Fig. 4:**
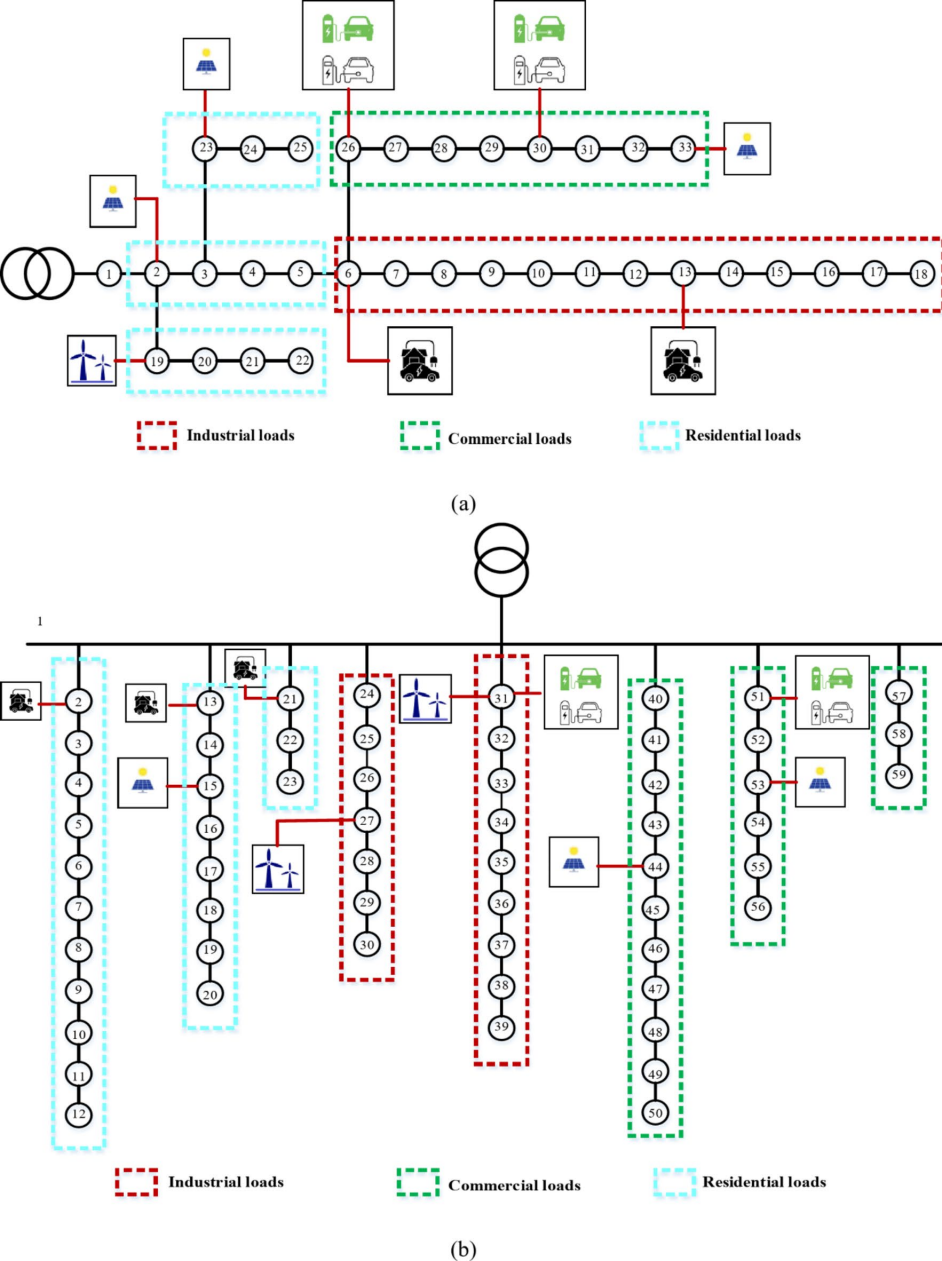
Single-line diagram of the suggested DNs: (**a**) IEEE 33-bus and (**b**) Cairo 59-bus.

**Fig. 5:**
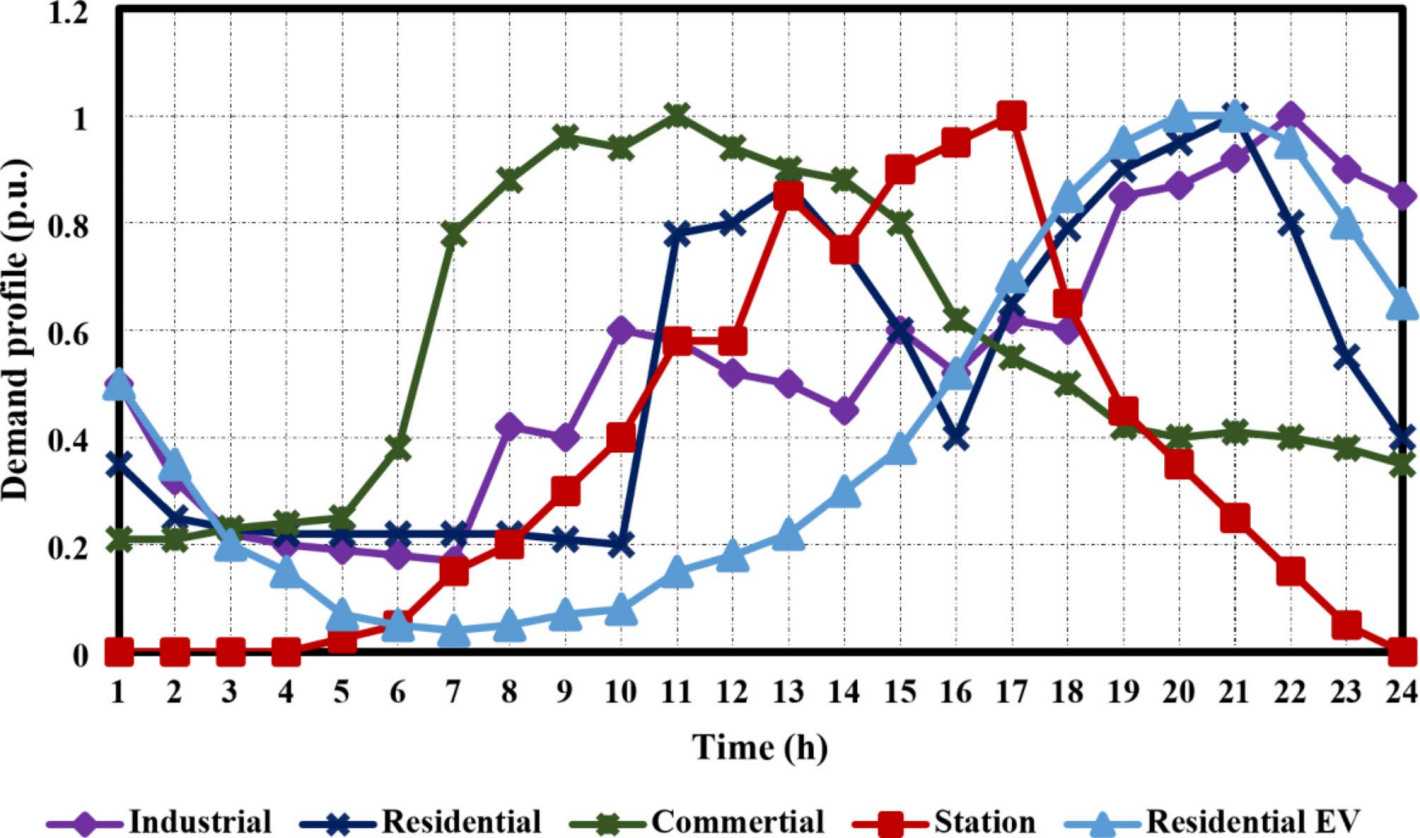
The daily profiles for EVs residential aggregators, charging stations, and load types.

The primary contribution of this study is to evaluate the combined DG-EV-HC while considering various time-varying load models, specifically commercial, residential, and industrial. In order to accomplish this, load type groups and system data from^37^ are used to classify the busloads of the IEEE 33-bus system into these three groups. The IEEE 33-bus system’s one-line diagram, which shows how the load kinds are categorized, is shown in Fig. 4a. A real 59-bus DN in Cairo, Egypt, was also selected as a test system in order to confirm the scalability of the suggested method^38^. This system has an apparent power base of 100 MVA, a base voltage of 22 kV, a peak overall consumption of 50.348 MW, and 21.448 MVAR. In the base-case scenario, the DN losses were estimated to be 391 kW in total when neither DGs nor EVs were integrated. For this system, busloads and feeder data were taken from^38^. This DN’s loads are categorized as follows: the first 23 loads are residential, the next 23 loads are industrial, and the last 23 loads are commercial. The single-line diagram of the Cairo 59-bus DN, which shows how the loads were categorized, is shown in Fig. 4b. In addition, the suggested load categories’ daily hour-by-hour load curves are shown in Fig. 5^39^. These graphs show how the load varies during the day. Table 2 lists the best places for RDGs, EV charging stations, and EVs aggregators in residential zones for the Cairo 59-bus and IEEE 33-bus systems, as shown also in Fig. 4. Regarding RDGs optimal locations, after multiple attempts to freely integrate the RDGs to load buses ranging from bus 2 to the last bus, these locations were chosen. The selected buses were found to be the most appropriate for hosting DGs through optimization under various configurations and conditions. An independent optimization model is executed for the optimal placement of RDGs in both the IEEE 33 bus and Cairo 59 bus systems, considering solely DGs and employing a single objective function aimed at maximizing DG-HC, with the variable of optimization being the placements of the DGs. Overvoltage was the only constraint on this optimization model. After that, this code was executed multiple times, and the most frequent optimal places were recorded. Note that there would be practical constraints and disadvantages if RDGs were assigned to every bus. Moreover, regarding the IEEE 33 bus, buses 26 and 30 were selected to host two EV charging stations. Moreover, busses 6 and 13 aggregate the residential area’s EV charging demand as stated in^25^. While authors^40^ concluded that the best number of EV charging stations and EV residential aggregators in Cairo’s 59-bus test system was, after eliminating the repeated buses, 2 stations and 3 aggregators, respectively. The optimal places for the stations were found to be buses 31 and 51, while buses 2,13, and 21 were the best places in the residential area to host the EV aggregators. The demand profiles depicted in Fig. 5 for EV residential aggregators and charging stations were obtained from^25^. These profiles were derived from the National Household Travel Survey (NHTS), which examined American public transportation users’ driving patterns.

**Table 2:** The optimal buses of the RDGs, EV charging stations, and EVs residential aggregators for both suggested systems.

Test system	DG/EV	Type	Location
IEEE 33-bus	DG_1_	PV-based (PV_1_)	Bus 2
	DG_2_	Wind-based	Bus 19
	DG_3_	PV-based (PV_2_)	Bus 23
	DG_4_	PV-based (PV_3_)	Bus 33
	EV_1_	Residential aggregator	Bus 6
	EV_2_	Residential aggregator	Bus 13
	EV_3_	Station	Bus 26
	EV_4_	Station	Bus 30
Cairo 59-bus	DG_1_	PV-based (PV_1_)	Bus 15
	DG_2_	Wind-based (WT_1_)	Bus 27
	DG_3_	Wind-based (WT_2_)	Bus 31
	DG_4_	PV-based (PV_2_)	Bus 44
	DG_5_	PV-based (PV_3_)	Bus 53
	EV_1_	Residential aggregator	Bus 2
	EV_2_	Residential aggregator	Bus 13
	EV_3_	Residential aggregator	Bus 21
	EV_4_	Station	Bus 31
	EV_5_	Station	Bus 51

### The proposed dynamic pricing and optimal load curves for systems under study

**Fig. 6:**
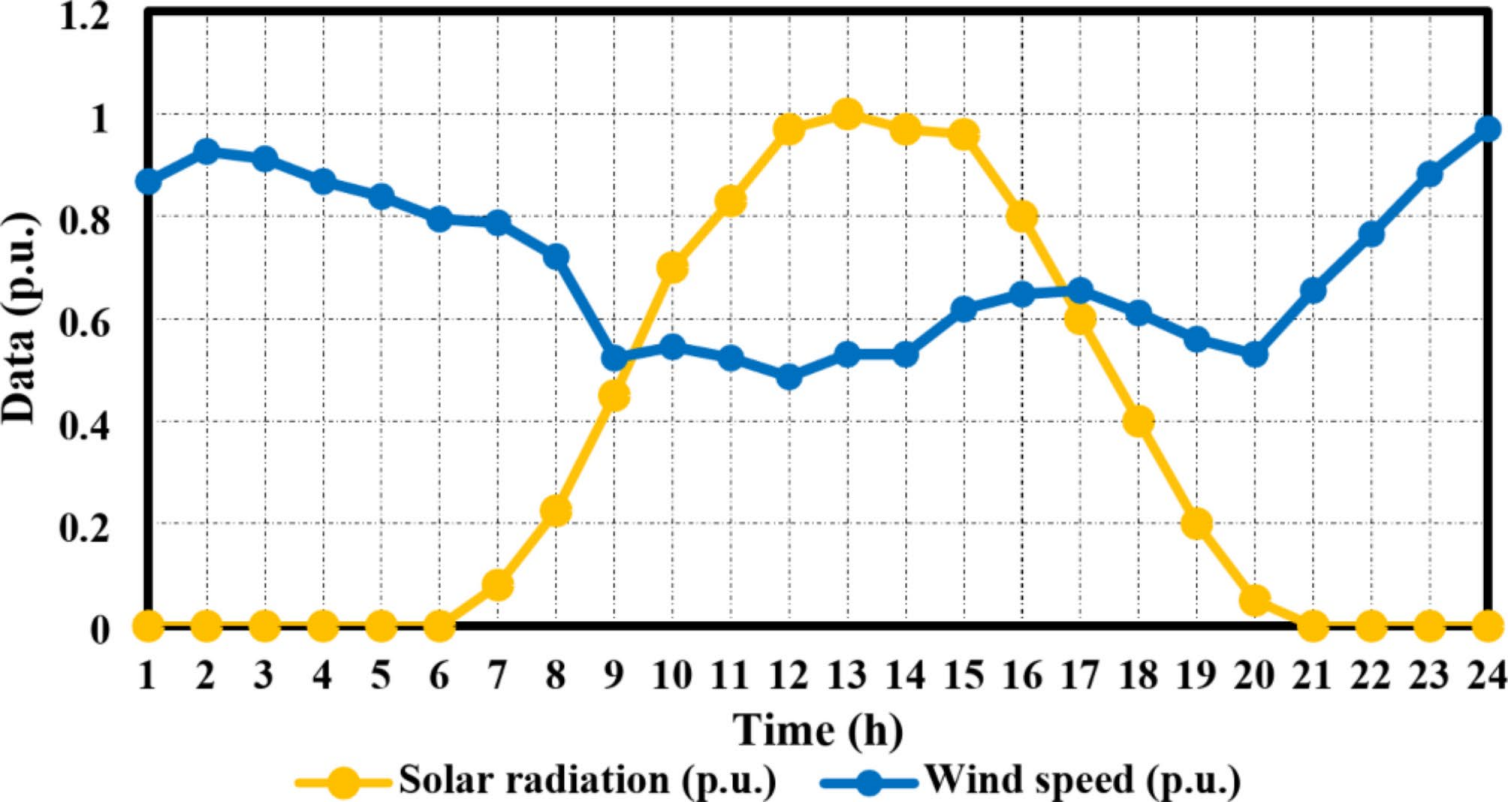
The day-ahead normalized solar radiation and wind speed.

The setup of dynamic pricing with the purpose of reducing overvoltage and improving HC is one of this paper’s major contributions. Based on the South Korean standard, the permitted voltage range is assumed between [0.91,1.04] per unit. Moreover, it is possible to enhance significantly the voltage-restricted combined DG-EV-HC by implementing this dynamic tariff. Wind speed data is taken from^27^, while solar radiation data is obtained from^41^ to enable the study and execution of the dynamic tariff. Figure 6 presents a graphical representation of wind speed and solar radiation data as normalized multipliers. The base value of solar radiation was set as 1000 w/m^2^, while the base wind speed value was 15 m/s.

**Fig. 7:**
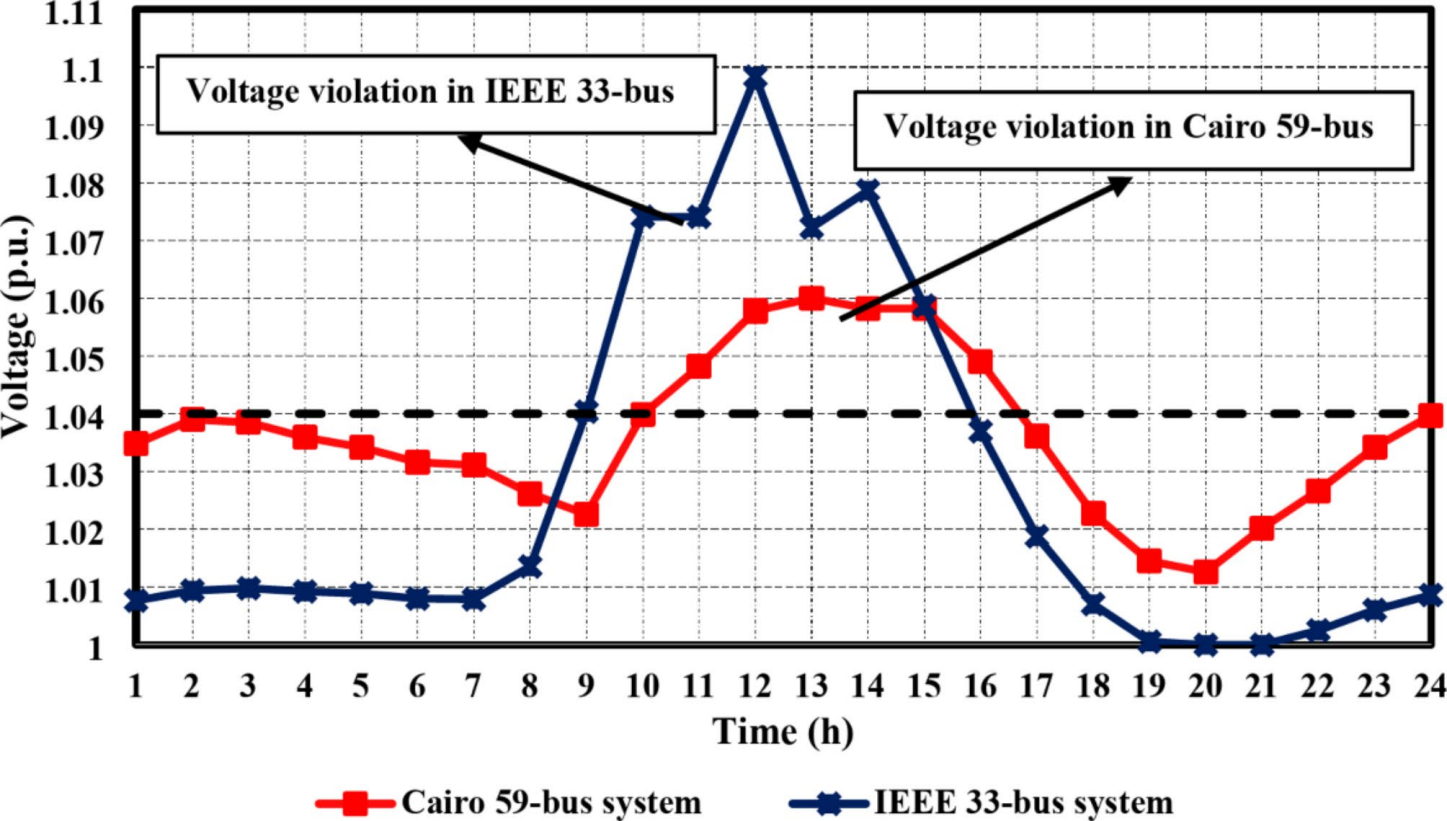
The maximum voltage recorded at each hour without considering any HC constraints for both suggested cases studied.

**Fig. 8:**
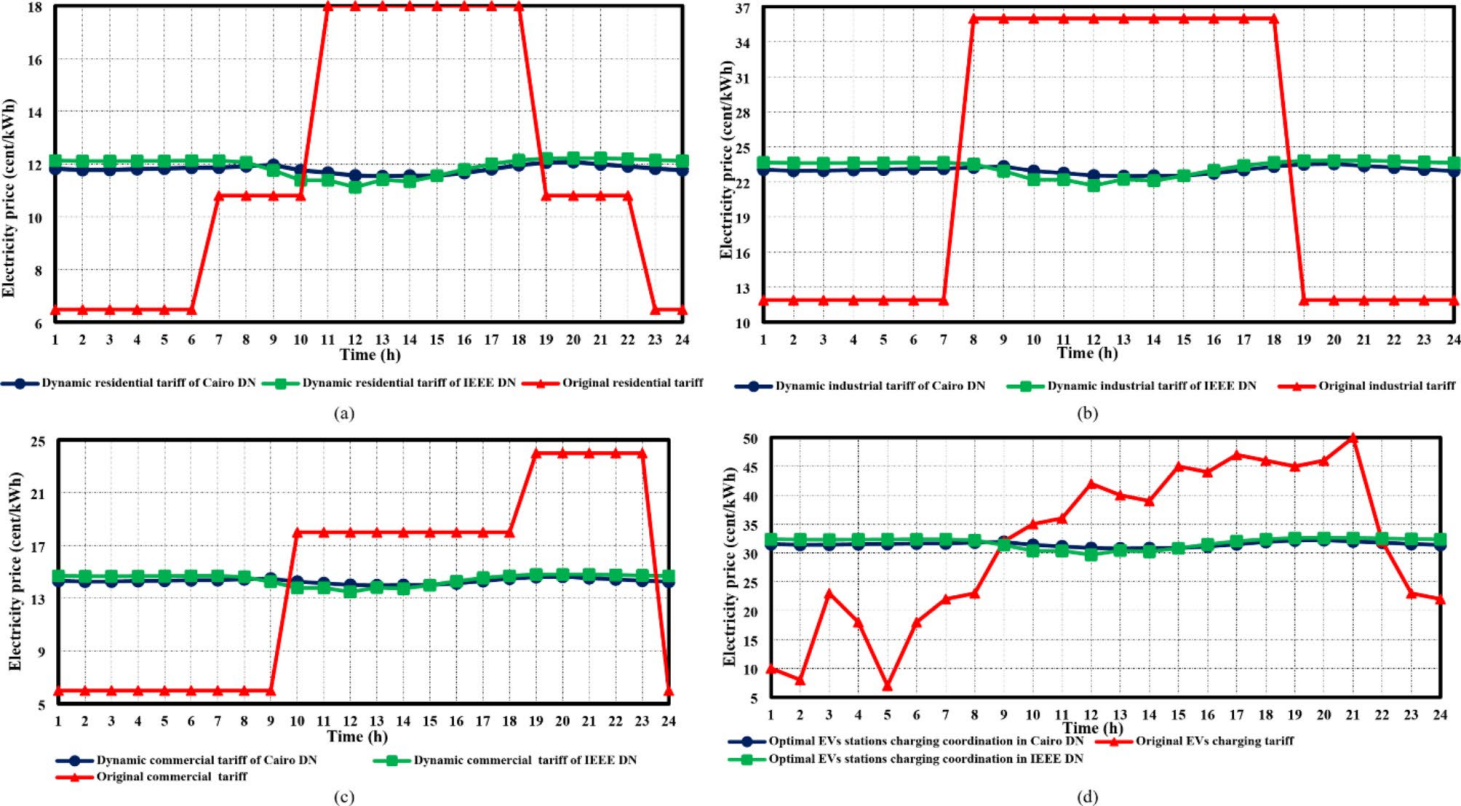
The proposed dynamic tariffs compared to the original tariffs for each load type in both DNs.

For both the IEEE 33-bus and Cairo 59-bus DNs, the maximum voltage observed at each hour ($\:{V}_{h}^{max}$) often surpasses the upper permissible limit ($\:{V}_{UB}$) when HC limitations are not considered, as seen in Fig. 7. The integrated DG units are assumed to inject their maximum active power at each hour, taking into account the variations in the original loads, EV charging profiles, wind speed, and solar radiation. The voltage profile for each hour is then calculated based on load flow, and the maximum voltage is determined. Equation (3) is used to compute the voltage rise percentage ($\:{V}_{rise}^{h}$), which is a measure of the degree of overvoltage. The dynamic tariff value for each hour *h* is then estimated by the DSO using an exponentially inverse-proportional relation with $\:{V}_{rise}^{h}$, as explained in Eq. (4). Figure 8 shows the suggested dynamic tariff ($\:{\pi\:}_{h}^{i}$) for various load categories during the day for both case studies. regarding residential, commercial, and industrial loads, the mean value of the old tariff ($\:{\stackrel{-}{\pi\:}}_{i}$) is considered, with the tariff values provided in^42^. However, EV residential aggregators’ charging costs follow the same residential tariff. The EV station charging cost is taken from^43^. The impact of implementing the suggested dynamic tariff function on the electricity tariff for all load categories in both employed DNs is displayed in Fig. 8. While optimizing hosting capacity was not a priority while defining the original tariff, the suggested dynamic tariffs have a lower value from *h* = 9 to *h* = 17 when there is a high availability of renewable energy. It ultimately reduces the possibility of a voltage rise issue by pushing each load’s home management system controller (HMSC) to shift higher load demand during this period of higher renewable availability. Moreover, Fig. 7 illustrates that the percentage of voltage rise in the IEEE 33-bus system was higher than that observed in the Cairo system during the period of *h* = 10 to *h* = 14. Consequently, as shown in Fig. 8, the dynamic tariff suggested for the IEEE 33-bus system was less than that of the Cairo system during the same period.

**Fig. 9:**
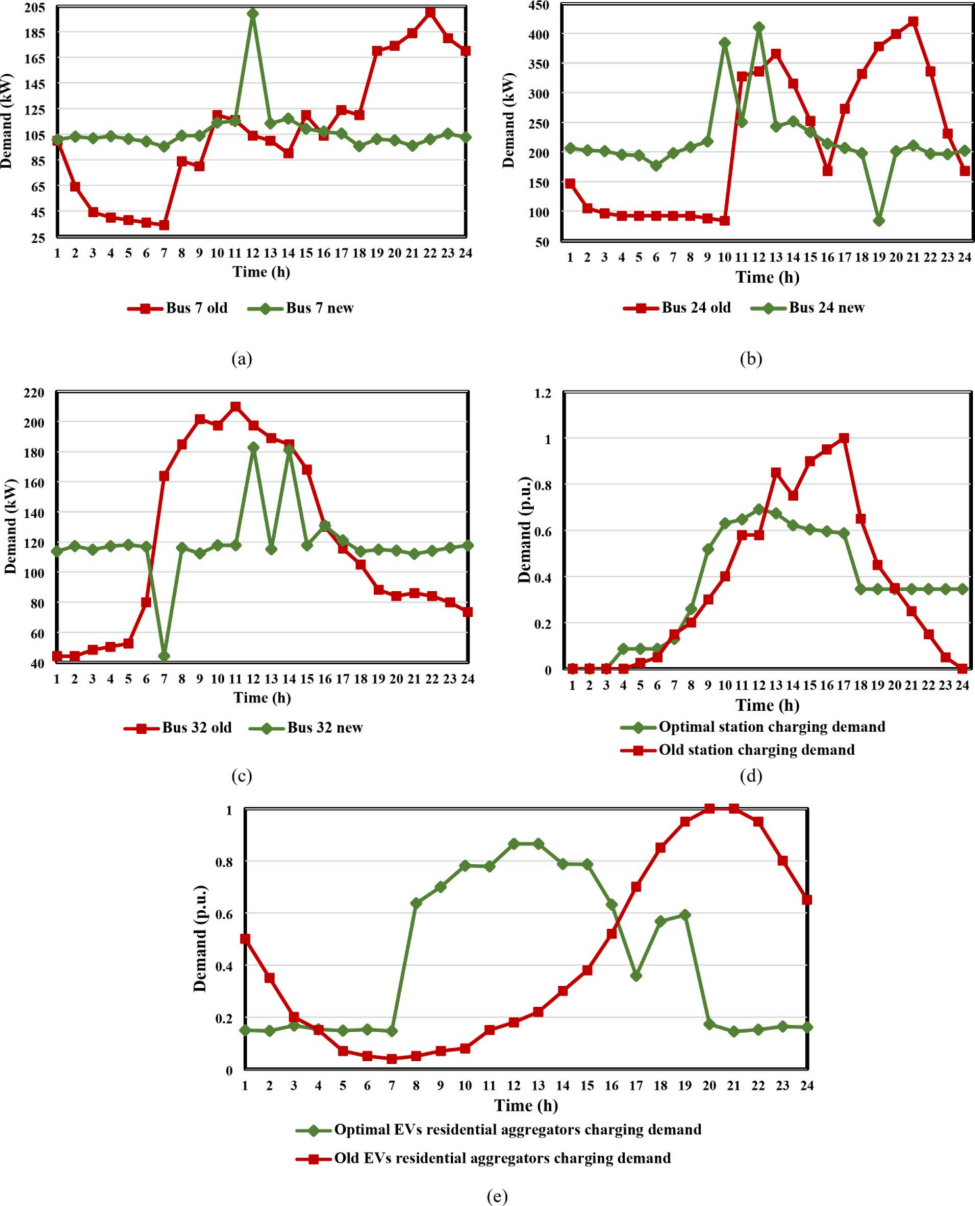
Comparison between the original and optimal load curves: (**a**) industrial, (**b**) residential, (**c**) commercial, (**d**) EVs stations, and (**e**) EVs residential aggregators for the IEEE 33-bus DN.

**Fig. 10:**
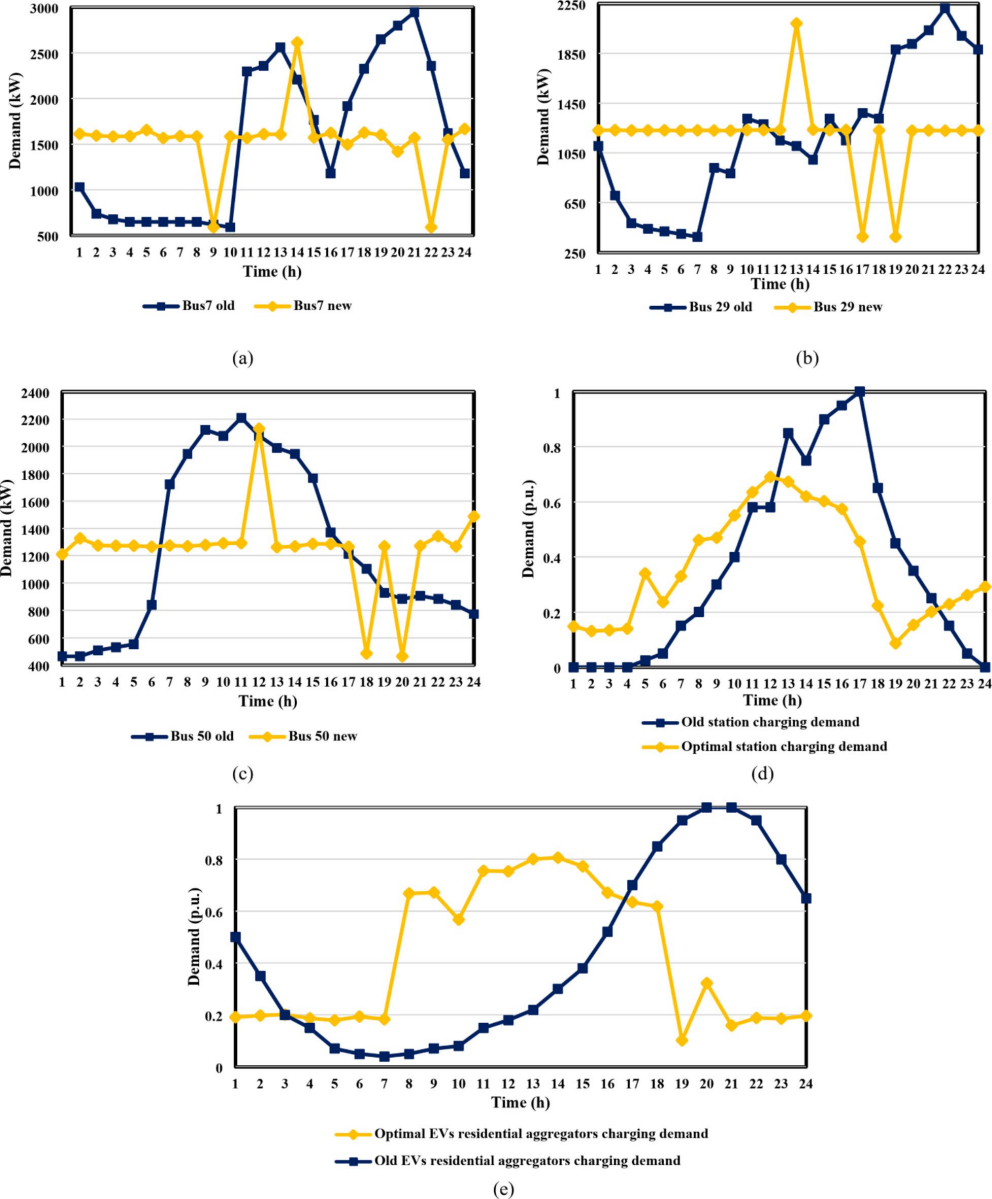
Comparison between the original and optimal load curves: (**a**) industrial, (**b**) residential, (**c**) commercial, (**d**) EVs stations, and (**e**) EVs residential aggregators for the Cairo 59-bus DN.

To minimize the total cost, each client applies the lower-stage optimization technique in their HMSC depending on the dynamic tariffs that the DSO provides, using Eqs. (17)–(19). In order to assess the combined DG-EV-HC with DR scenarios in the case studies, this optimization procedure produces optimal load curves for each load and optimal EV charging coordination. The impact of the suggested DR program on the load curves is demonstrated in Figs. 9 and 10, which highlight the highest loads in the residential, commercial, and industrial load categories, as well as the charging profile of EVs at stations and domestic aggregators. Regarding IEEE 33-bus DN, Fig. 9(a) illustrates the modification in the demand curves for the highest industrial load (placed at bus 7) in accordance with the proposed dynamic pricing and voltage rise percentages. Also, Fig. 9(b) presents the shift that occurred to the peak interval for the highest residential load from *h* = 21 to *h* = 12, which corresponded to the most severe voltage rise recorded. Moreover, the greatest commercial load on bus 32 was shown to have shifted from *h* = 11 to *h* = 12 in Fig. 9(c), which significantly contributed to reducing the voltage rise problem, which was at its worst value during this hour. Figure 9d, e illustrate the optimal charging coordination for both EV charging stations and EV aggregators, taking a 25 MW base value for each of them. As demonstrated in both figures, the highest charging demands were focused on the interval of *h*= [9,15], at which the voltage rise issue was noticed. Finally, Fig. 10 shows how the proposed dynamic tariff modifies the load curves and EV charging patterns.

### Results and discussion

The article’s primary objective is to study the effect of an optimization-based dynamic pricing DR approach mixed with a Volt/VAR optimal control of SIs on the combined DG-EV-HC. In addition, the impact of integrating EVs to the DN on the estimated DG-HC is studied in this work. In order to achieve these objectives, the work in the paper is structured into four cases. These cases are explained as follows:

- Case I estimates the optimal DG-HC disregarding EVs aggregators and enhancement techniques.
- Case II estimates the optimal DG-HC and EV-HC simultaneously such that the effect of integrating EVs on DG-HC is illustrated.
- Case III estimates the optimal DG-HC and EV-HC simultaneously, considering only the proposed DR program.
- Case IV estimates the optimal DG-HC and EV-HC simultaneously, considering both the DR program and SIs Volt/VAR control.

Moreover, the effect of suggested multi-objective function weighting factors on enhancing the combined DG-EV-HC while minimizing system losses is studied in this research. It was found through numerous trials that larger EV-HC values allow higher DG integration without violating the safe operation of the system. DG-HC and EV-HC levels were at their maximum when $\:{w}_{1}=0.5,\:{w}_{2}=0.5,\:{w}_{3}=0$. The total active power loss, however, considerably exceeded the base case value during multiple hours, particularly those with higher wind speed and solar irradiance. This demonstrated how crucial it was to incorporate the total active power loss index into the multi-objective function in order to attain an acceptable optimal DG-HC and EV-HC, provided that the total active power loss stayed below the base case. Selecting the appropriate objective function’s weighing factors carefully was essential to striking this compromise. After several trials, the values of $\:{w}_{1}$, $\:{w}_{2}$, and $\:{w}_{3}$ were arbitrarily altered from 0 to 1. Only the trials resulting in power loss outcomes that did not exceed the base case were accepted. Table 3 offers the simulation parameters that were considered in this work.

In addition, the weighting factors that were obtained showed an average value of almost 0.35 for the DG-HC index ($\:{w}_{1}$), 0.35 for the EV-HC index ($\:{w}_{2}$), and 0.3 for the term of total active power loss ($\:{w}_{3}$). This highlights how crucial it is to strike an appropriate balance between enhancing the DG-EV hosting capacity and lowering the overall active power loss to achieve a desirable and optimal result.

Additionally, the effect of changing the population number and maximum number of iterations is studied by performing multiple trials. It was noticed that increasing the maximum number of iterations and population numbers above 300 and 30, respectively, consumes substantial computational time and achieves trivial improvement in the proposed multi-objective function value.

**Table 3:** Simulation parameters used in the investigations.

Parameter	Value (unit)
Particles number	30
Maximum number of iterations	300
$\:{w}_{1},{w}_{2},\:and\:{w}_{3}$	0.35, 0.35 and 0.3
$\:{V}_{min}$and $\:{V}_{max}$	0.91 and 1.04
$\:{G}_{c}$ and $\:{G}_{std}$	150 and 1000 (W/m^2^)
$\:{V}_{cut-in}$	4 (m/s)
$\:{V}_{cut-out}$	25 (m/s)
$\:{V}_{rated}$	15 (m/s)
$\:{P}_{WT}^{max}$ and $\:{P}_{PV}^{max}$ (IEEE 33 bus)	25 (MW)
$\:{P}_{W}^{max}$ and $\:{P}_{PV}^{max}$ (Cairo 59 bus)	80 (MW)
$\:{EV}_{r}^{max},\:{EV}_{c}^{max}$(IEEE 33 bus)	25 (MW)
$\:{EV}_{r}^{max},\:{EV}_{c}^{max}$(Cairo 59 bus)	30 (MW)
$\:{Q}_{max}$ (IEEE 33 bus)	10 (MVAR)
$\:{Q}_{max}$ (Cairo 59 bus)	15 (MVAR)
$\:{m}_{1}^{min}$ and $\:{m}_{1}^{max}$	-25 and − 5
$\:{m}_{2}^{min}\:$and $\:{m}_{2}^{max}$	-30 and − 15

**Fig. 11:**
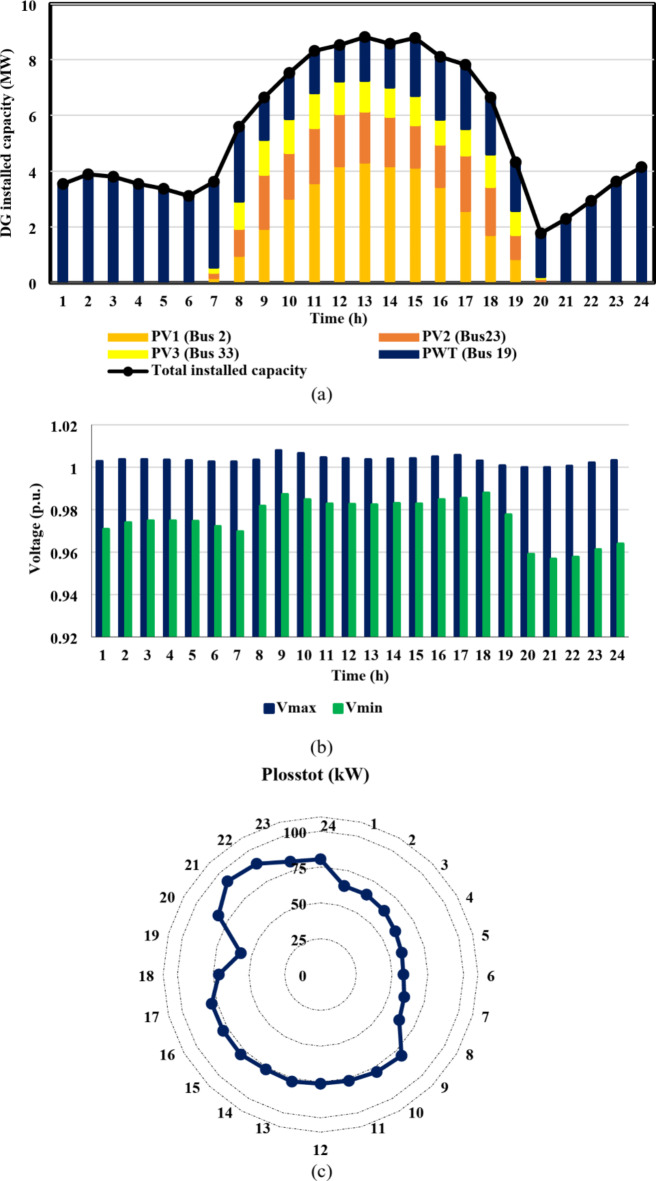
IEEE 33-bus results through case 1 using the EO algorithm: (**a**) maximum installed capacity for every DG unit for the duration of the day, (**b**) greatest and lowest bus voltages throughout the entire day, and (**c**) power loss through the whole day.

**Fig. 12:**
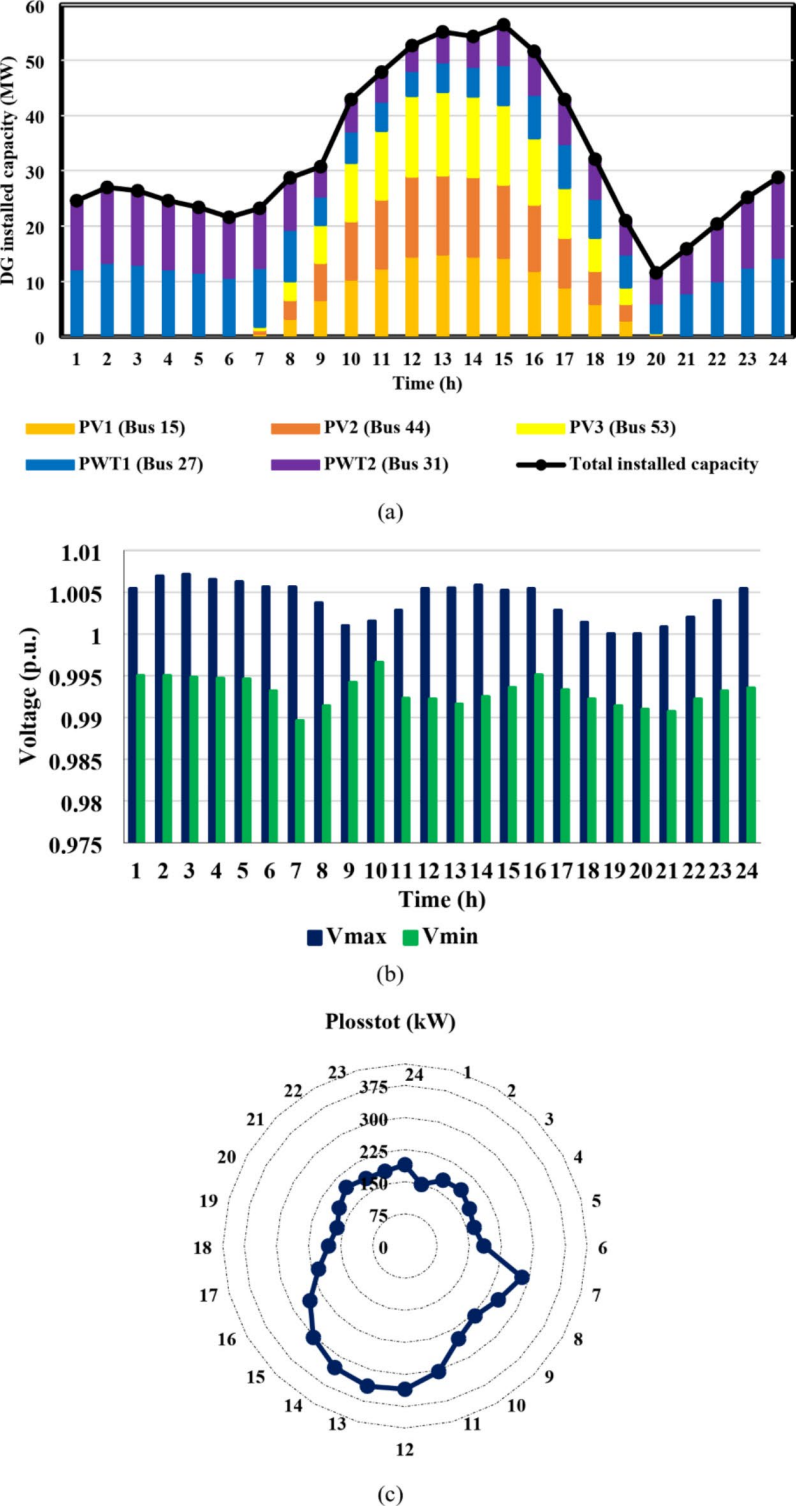
Cairo 59-bus results through case 1 using the EO algorithm: (**a**) maximum installed capacity for every DG unit for the duration of the day, (**b**) greatest and lowest bus voltages throughout the entire day, and (**c**) power loss throughout the whole day.

From the results of case I, shown in Figs. 11 and 12, the IEEE 33-bus and Cairo 59-bus DNs were found to have the ability to host hourly optimal installed capacities of 5.5 MW and 33 MW, on average. The highest observed installed capacity values were 8.8 MW at h = 13 For the IEEE 33-bus and 56.4 MW at h = 15 for the Cairo 59-bus, respectively. Furthermore, as shown by Fig. 11(b) and 12(b), all bus voltages for both systems during the duration of the day were within the allowed ranges because neither the maximum bus voltage nor the minimum bus voltage exceeded the upper or lower constraints. Moreover, as Fig. 11(c) and 12(c) show, the base case values of the IEEE 33-bus and Cairo 59-bus were not exceeded by the total active power loss. The loss for a Cairo 59-bus was estimated to be among [149,339] kW, whereas the loss for the IEEE 33-bus was in the range of [59,92] kW.

**Fig. 13:**
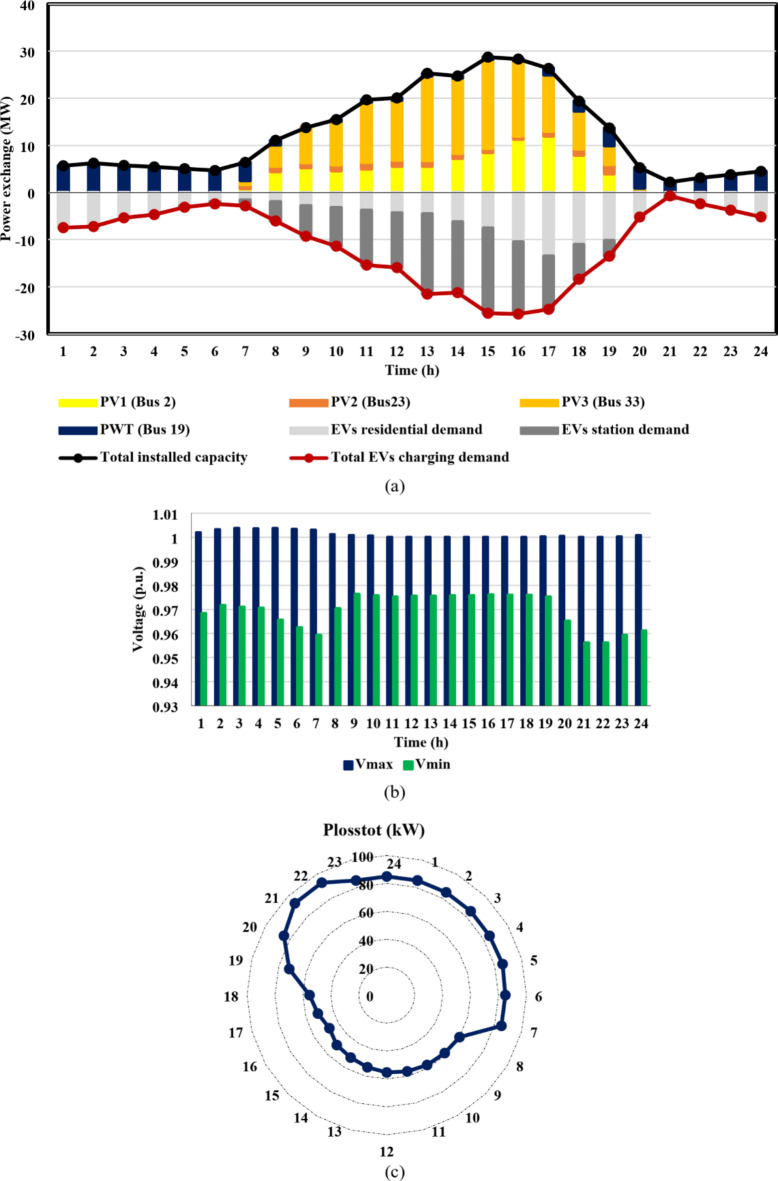
IEEE 33-bus results through case 2 using the EO algorithm: (**a**) maximum installed capacity for every DG unit and maximum EV demand of both aggregator types for the duration of the day, (**b**) greatest and lowest bus voltages throughout the entire day, and (**c**) power loss through the whole day.

**Fig. 14:**
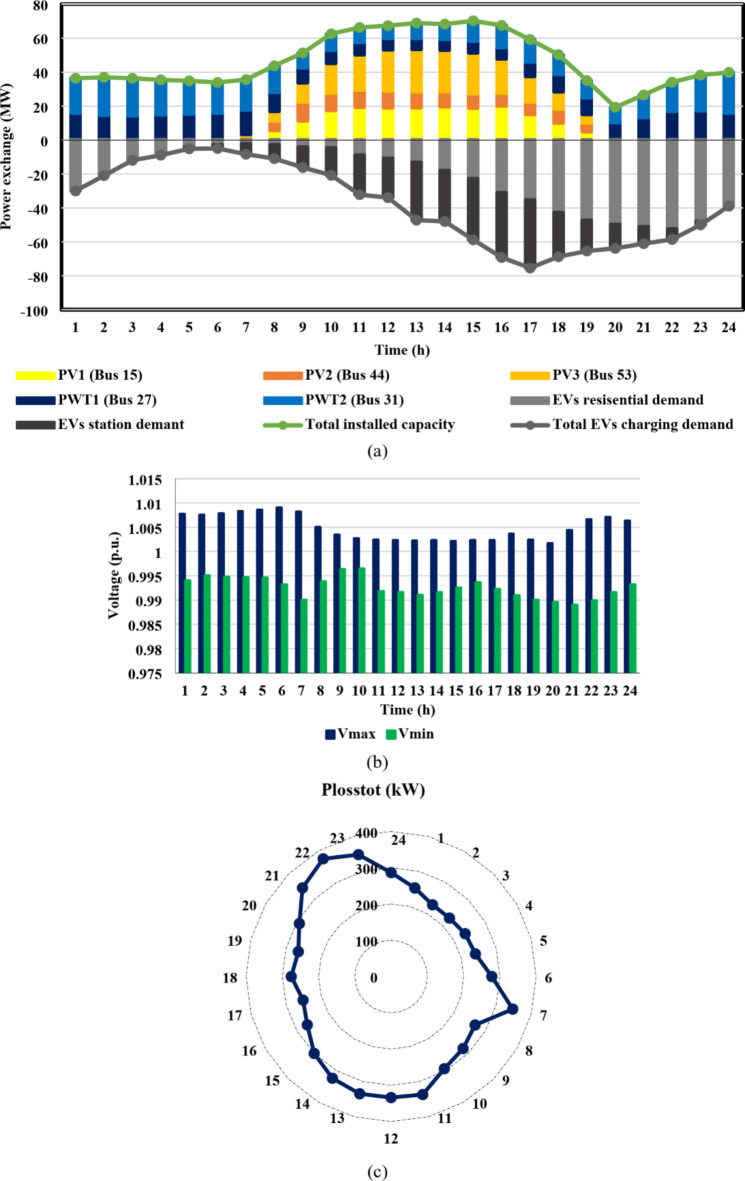
Cairo 59-bus results through case 2 using the EO algorithm: (**a**) maximum installed capacity for every DG unit and maximum EV demand of both aggregator types for the duration of the day, (**b**) greatest and lowest bus voltages throughout the entire day, and (**c**) power loss through the whole day.

Furthermore, case II reveals the impact of EV integration on optimal DG-HC estimation. Using the suggested multi-objective function, Fig. 13(a) and 14(a) demonstrated that the IEEE 33-bus and Cairo 59-bus, respectively, can host up to 10.8 MW and 37.9 MW of EV aggregator charging demands on average, without taking any enhancing approaches into account. Because of the massive EVs charging demand drawn from the DN, it was possible to host higher RDG without violating the system’s limits. As a result, for the IEEE 33 bus, the mean value of the optimal RDG installed capacity increased by almost 133%, from 5.5 MW to 12.8 MW. In addition, there was a significant rise in the average value of the optimal RDG installed, rising from 33 MW to 46.6 MW, or about 41.2%. As seen in Fig. 13(c) and 14(c), despite the massive values of DG-HC and EV-HC that were recorded, the multi-objective function’s consideration of the power loss index and the appropriate choice of weighting factors prevented the total active losses from exceeding the base case value for the whole day. The minimum value of total active power loss for the IEEE 33-bus was recorded at h = 16 when it was around 48 kW, and the maximum was recorded at h = 21 when it was 93 kW. Concerning the Cairo 59-bus, the lowest *P*_*loss*_ was 228 kW at h = 3, while the highest *P*_*loss*_ was about 375 kW at h = 22. Additionally, Fig. 13(b) and 14(b) show that all bus voltages were accepted.

**Fig. 15:**
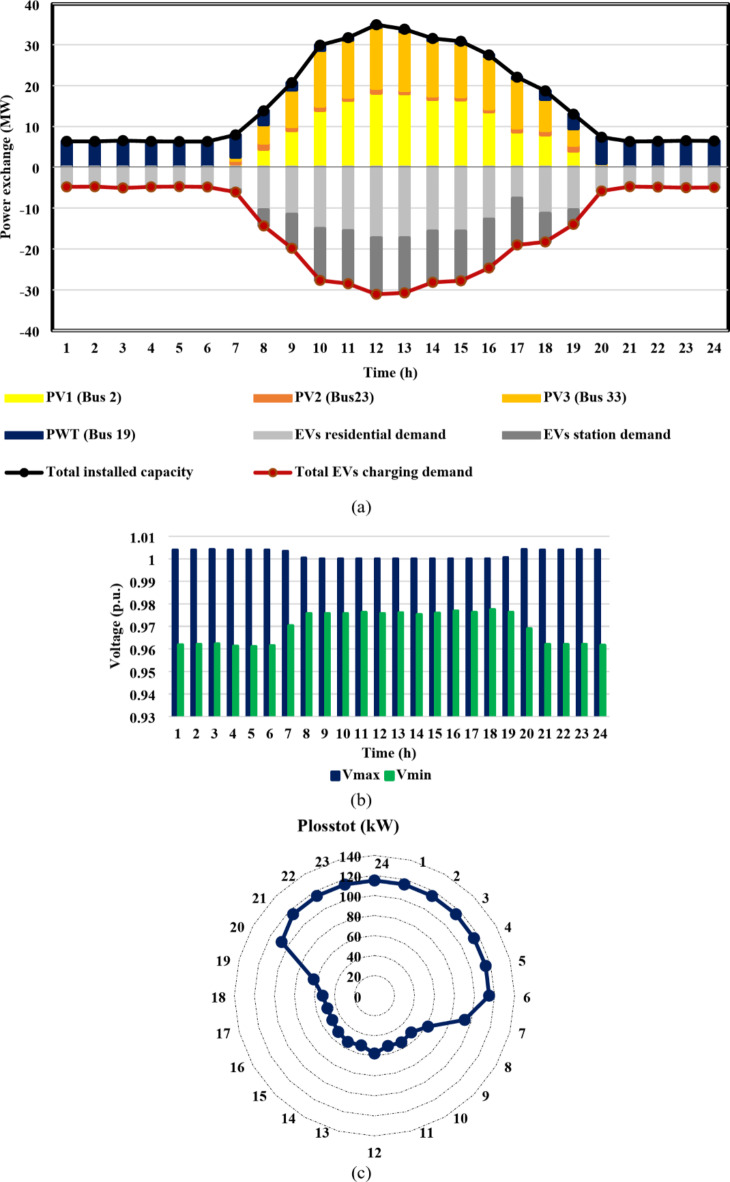
IEEE 33-bus results through case 3 using the EO algorithm: (**a**) maximum installed capacity for every DG unit and maximum EV demand of both aggregator types for the duration of the day, (**b**) greatest and lowest bus voltages throughout the entire day, and (**c**) power loss through the whole day.

**Fig. 16:**
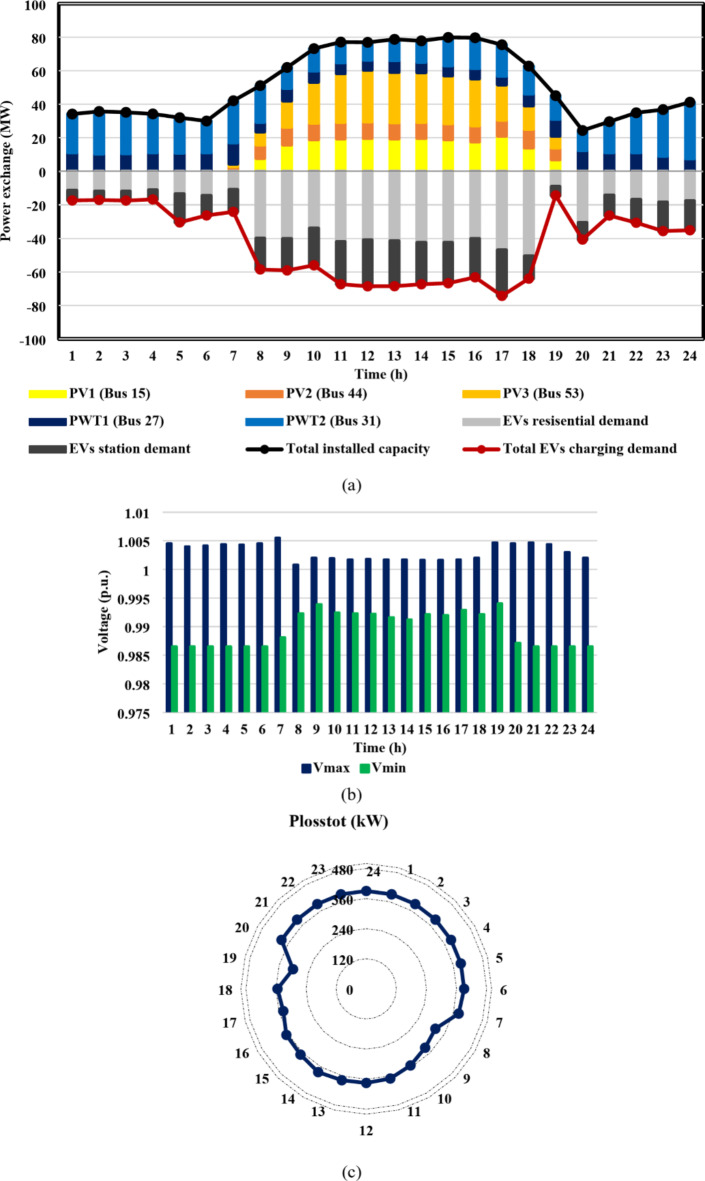
Cairo 59-bus results through case 3 using the EO algorithm: (**a**) maximum installed capacity for every DG unit and maximum EV demand of both aggregator types for the duration of the day, (**b**) greatest and lowest bus voltages throughout the entire day, and (**c**) power loss through the whole day.

The results of the third case strongly indicate the effectiveness of the suggested DR approach in enhancing the combined DG-EV-HC. The proposed DR program provides a significant increase in the mean value of the optimal installed capacity of the IEEE 33-bus DN throughout the day, from 12.8 MW to 16.2 MW, which is almost 27% higher, as shown in Fig. 15(a) compared with Fig. 13(a). Moreover, the Cairo 59-bus DN’s mean optimal installed capacity increased by 12.5%, from 46.6 MW to 52.2 MW, as illustrated in Fig. 14(a) compared with Fig. 16(a). The remarkable positive effect of the proposed DR program on EV-HC enhancement is revealed clearly in Fig. 15(a) versus Fig. 13(a) for IEEE 33-bus and Fig. 16(a) versus Fig. 14(a) for Cairo 59-bus. The mean value of the total EVs charging load through the whole day increased dramatically from 10.8 MW to 14.4 MW, almost 34% improvement. Regarding Cairo 59-bus, its ability to host EV charging demand improved from 37.9 MW to 43.7 MW, about 15.5% higher. In addition, the safe operation of both DNs despite these higher RDG penetration and EVs demands is illustrated in Fig. 15(b), 15(c), 16(b) and 16(c). Figure 15(c) illustrates that for the IEEE 33-bus system, all recorded *P*_*loss*_ values fell within the range of [49,115] kW. In contrast, Fig. 16(c) displays *P*_*loss*_ values for the Cairo 59-bus system, which ranged from [303,390] kW. Furthermore, all bus voltages for both systems were accepted during the entire day as illustrated in Fig. 15(b) and 16(b).

**Fig. 17:**
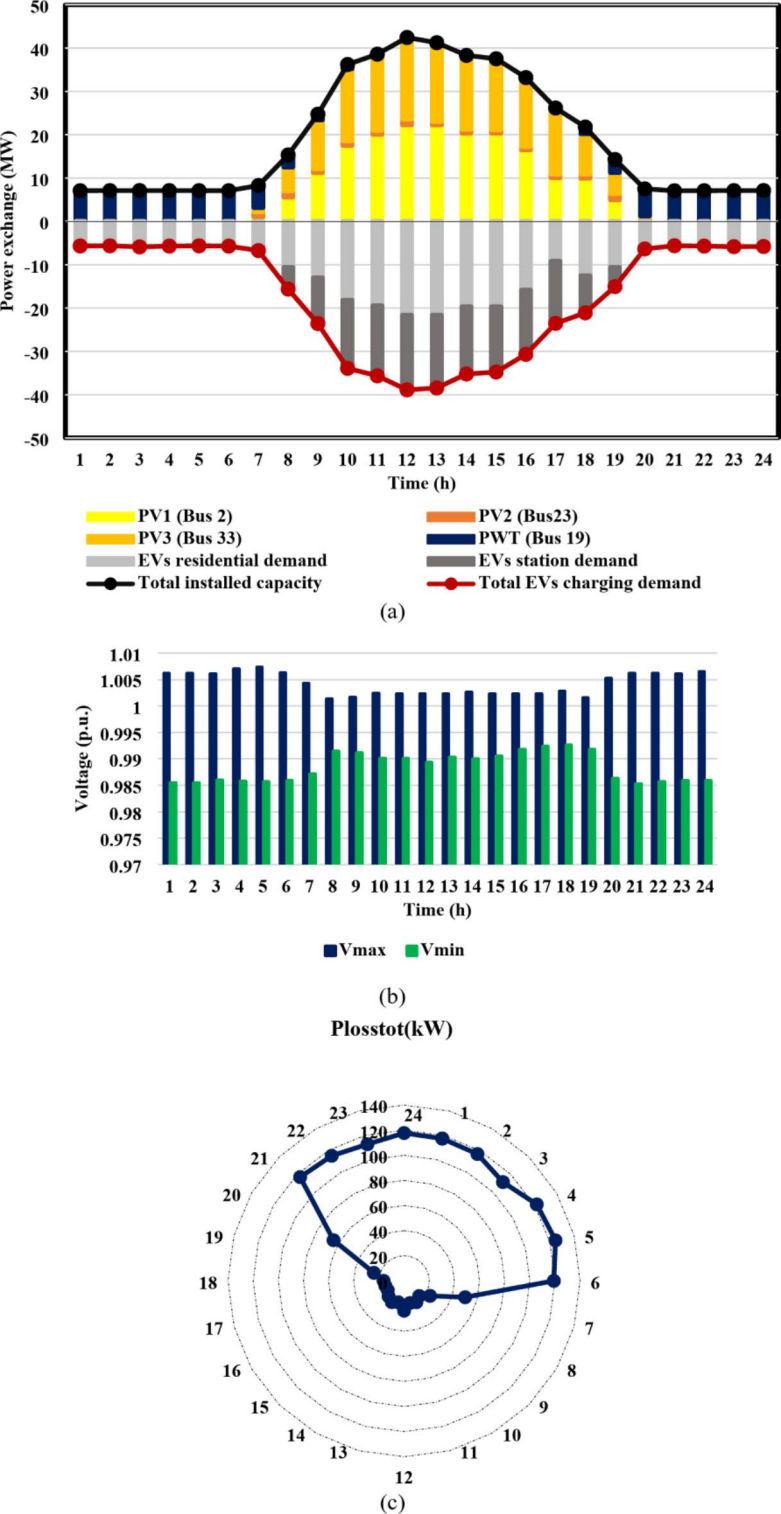
IEEE 33-bus results through case 4 using the EO algorithm: (**a**) maximum installed capacity for every DG unit and maximum EV demand of both aggregator types for the duration of the day, (**b**) greatest and lowest bus voltages throughout the entire day, and (**c**) power loss through the whole day.

**Fig. 18:**
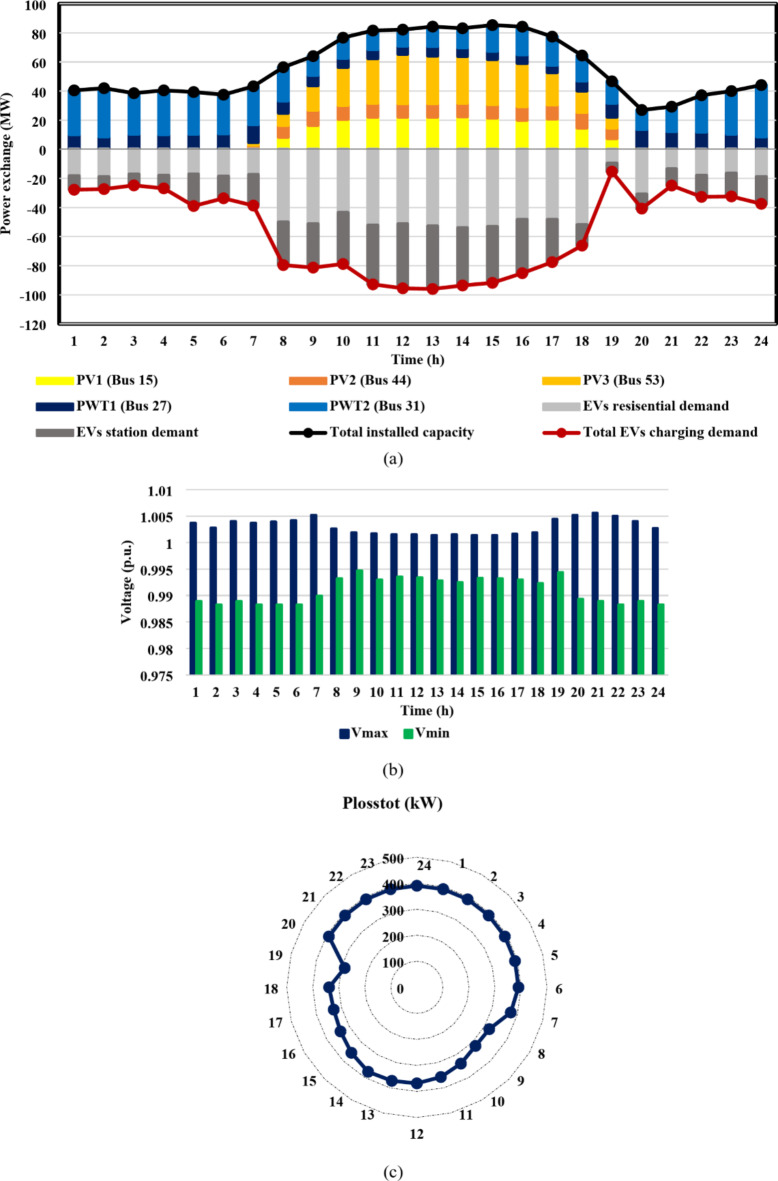
Cairo 59-bus results through case 4 using the EO algorithm: (**a**) maximum installed capacity for every DG unit and maximum EV demand of both aggregator types for the duration of the day, (**b**) greatest and lowest bus voltages throughout the entire day, and (**c**) power loss through the whole day.

**Fig. 19:**
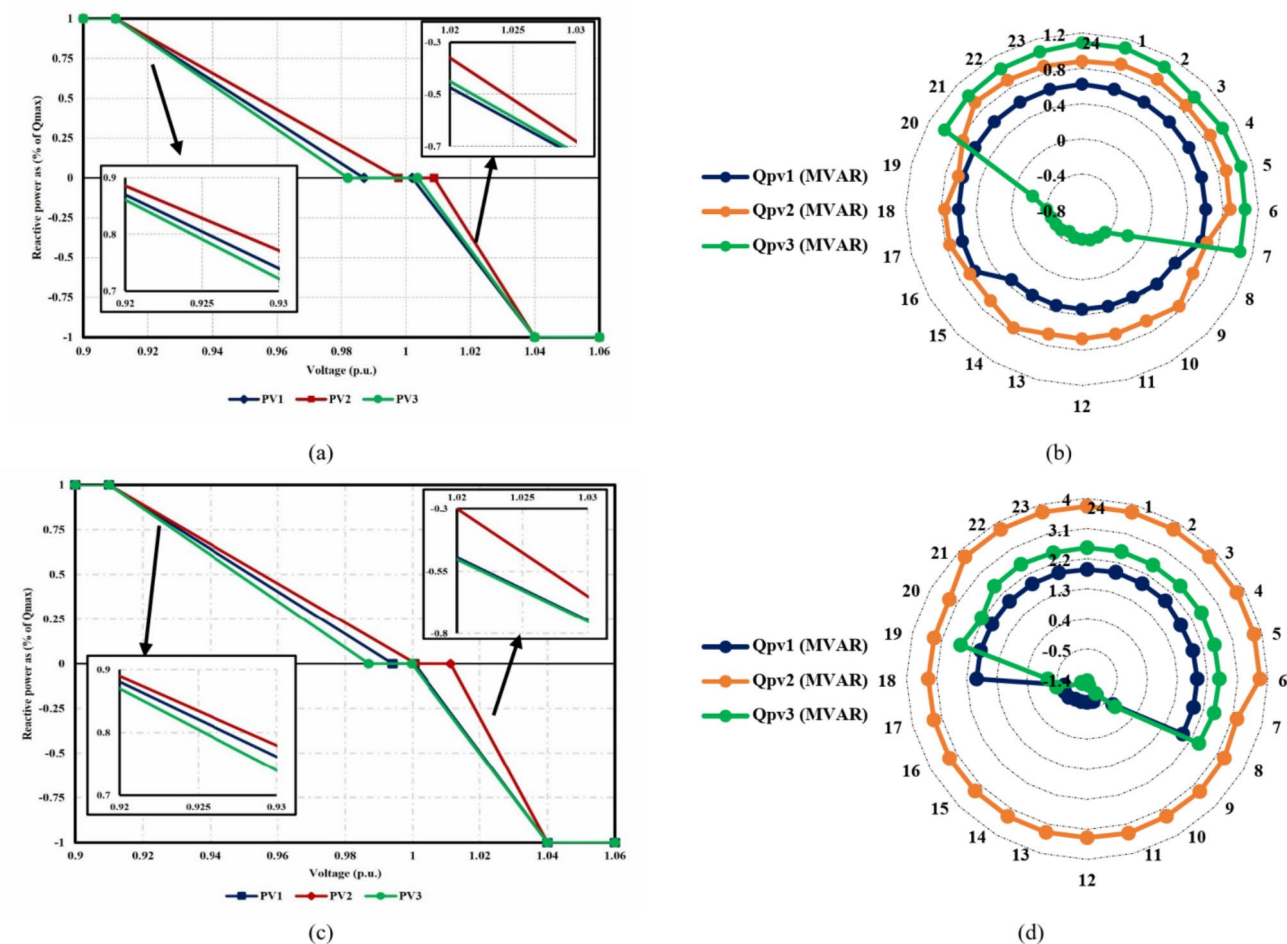
SIs findings for both systems through case 4: (**a**) the IEEE 33-bus optimal voltage/VAR control settings (**b**) the IEEE 33-bus reactive power profile (**c**) the Cairo 59-bus optimal voltage/VAR control settings (**d**) the Cairo 59-bus reactive power profile.

**Fig. 20:**
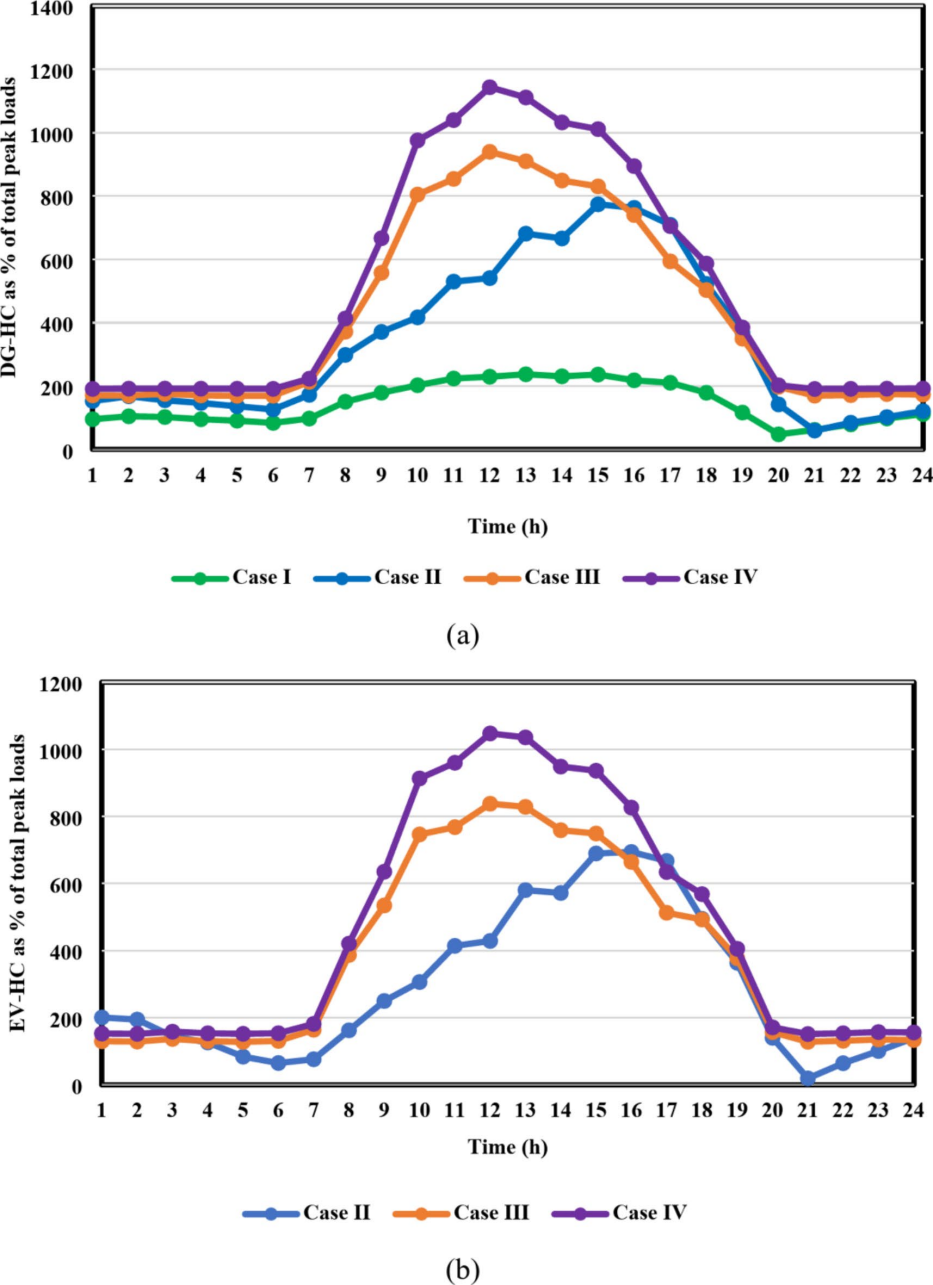
Comparison between the optimal DG-HC and EV-HC in the four cases for IEEE 33-bus system: (**a**) DG-HC and (**b**) EV-HC.

**Fig. 21:**
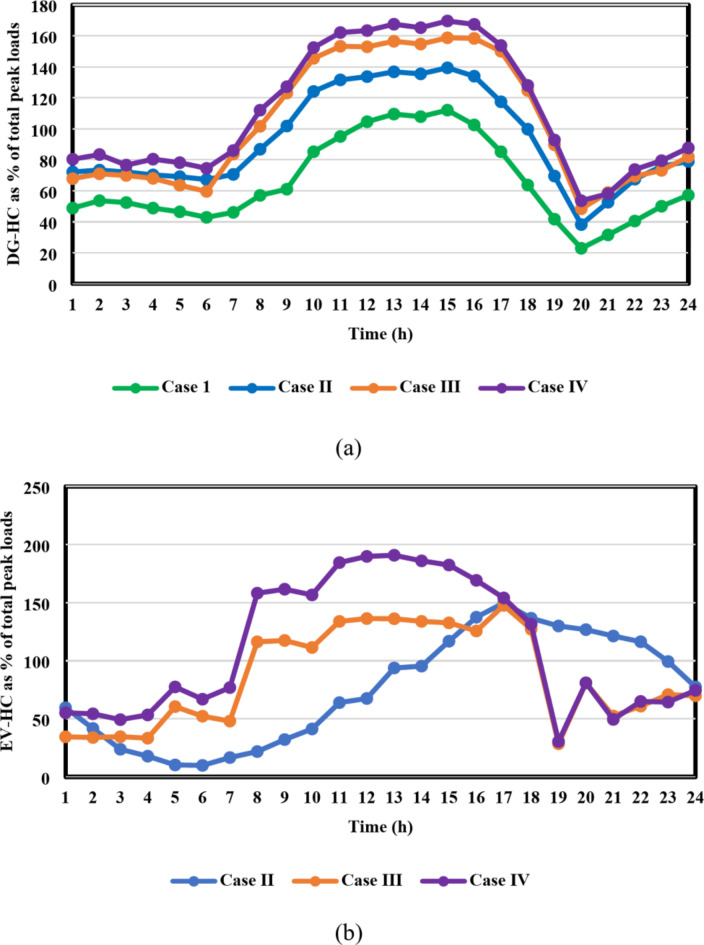
Comparison between the optimal DG-HC and EV-HC in the four cases for Cairo 59-bus system: (**a**) DG-HC and (**b**) EV-HC.

The fourth-case findings illustrate the superiority of the proposed synergetic enhancement technique, which combines DR and Volt/VAR control. Compared with case II where no enhancement techniques applied, the hybrid DR-SIs control approach greatly increases the average value of the optimal RDG installed capacity, of the IEEE 33-bus and Cairo 59-bus DNs throughout the day, as seen in Fig. 17(a) and 18(a). The value rose from 12.8 MW to 19.1 MW, a 49.2% rise, and from 46.6 MW to 56.1 MW, a roughly 20.4% rise, respectively. Stated alternatively, the mean value of the DG-HC rose from 92.6% (unenhanced) to 111.4% (fully enhanced) of the total peak loads for the Cairo 59-bus and from 344.5% (unenhanced) to 514.1% (fully enhanced) of the total peak loads for the IEEE 33-bus. Not only did the DG-HC get a great share of improvement, but the EV-HC also. For IEEE 33-bus, the average value of the entire day’s EV charging load jumped significantly from 10.8 MW to 17.4 MW or an improvement of almost 61.2%. When it comes to the Cairo 59-bus, its capacity to host EV charging demand increased from 37.9 MW to 55.9 MW or 47.5% more. Stated alternatively, the mean value of the EV-HC increased for the Cairo 59-bus from 75.3% (unenhanced) to 111% (completely improved) of the total peak loads and for the IEEE 33-bus from 290.7% (unenhanced) to 468.4% (completely improved) of the total peak loads. Figure 17(c) and 18(c) confirm the safe operation of the DNs regarding the total active power loss. *P*_*loss*_ in IEEE 33-bus ranged in the interval [15,125] kW, while [287,390.5] kW was the range of *P*_*loss*_ in Cairo 59-bus. Additionally, Voltage constraints were confirmed through Fig. 17(b) and 18(b). In addition, the optimal Volt/VAR control settings and optimal reactive power output for the SIs are depicted in Fig. 19. Finally, a comparison between the hourly DG-HC and EV-HC levels throughout the whole day, as a percentage of total peak loads, through the proposed cases for both test systems is illustrated in Figs. 20 and 21.

### Assessment of the EO optimizer’s performance

The EO performed significantly better in solving the given problem, as seen by the findings shown in Tables 4 and 5. The EO’s performance is compared against other optimization algorithms, including golden jackal optimization (GJO)^44^, particle swarm optimization (PSO)^45^, and zebra optimization algorithm (ZOA)^46^. The findings are based on 10 different runs, with the search agents and maximum iteration numbers chosen at 30 and 300, respectively. Tables 4 and 5 display the convergence outcomes, including the best and worst obtained results over the 10 runs of the selected four optimizers for the IEEE 33-bus DN at h = 13 and the Cairo 59-bus at *h* = 15 through case II, case III, and case IV, respectively. Unlike the other cases, which include all terms under study, i.e., DG-HC, EV-HC, and power loss, Case I disregarded EV-HC in multi-objective function. As a result, the multi-objective function was defined only in terms of DG-HC and power loss. Therefore, case I wasn’t considered in the convergence results. In addition, the amount of time needed to complete the best run is also stated. Finally, a robustness factor “*α*” is defined in this article as explained in Eq. (30). It can be calculated for each optimizer by dividing the number of times reached the best solution “$\:{N}_{best}$” over the total number of runs “$\:{N}_{tot}$”.

30 $$\:\alpha\:=\frac{{N}_{best}}{{N}_{tot}}$$

Regarding IEEE 33-bus results, GJO converged at worse values of the objective function through certain runs (two runs), namely 0.2851 at case III and 0.1939 at case IV, which are excessively distant from the best solution found by other optimizers. When compared to other optimizers, ZOA in case IV converged to the worst convergence results across all runs. The objective function obtained at the best run was 0.12076, which is over 16% worse than the best objective function that EO could achieve. In all cases, PSO converged to approved outcomes. Through cases II and III, the best run consumed the least amount of execution time; through case IV, it consumed the greatest execution time.

With regard to the Cairo 59-bus, GJO only achieved the best objective function twice in case IV and demonstrated a significant variation between the optimal values in the best and worst values. In terms of the time of executing one complete run in all cases, PSO ranked best; however, EO outperformed it regarding the obtained objective function values. ZOA showed good convergence results in all three cases, but in each case, the complete run took the longest to finish.

**Fig. 22:**
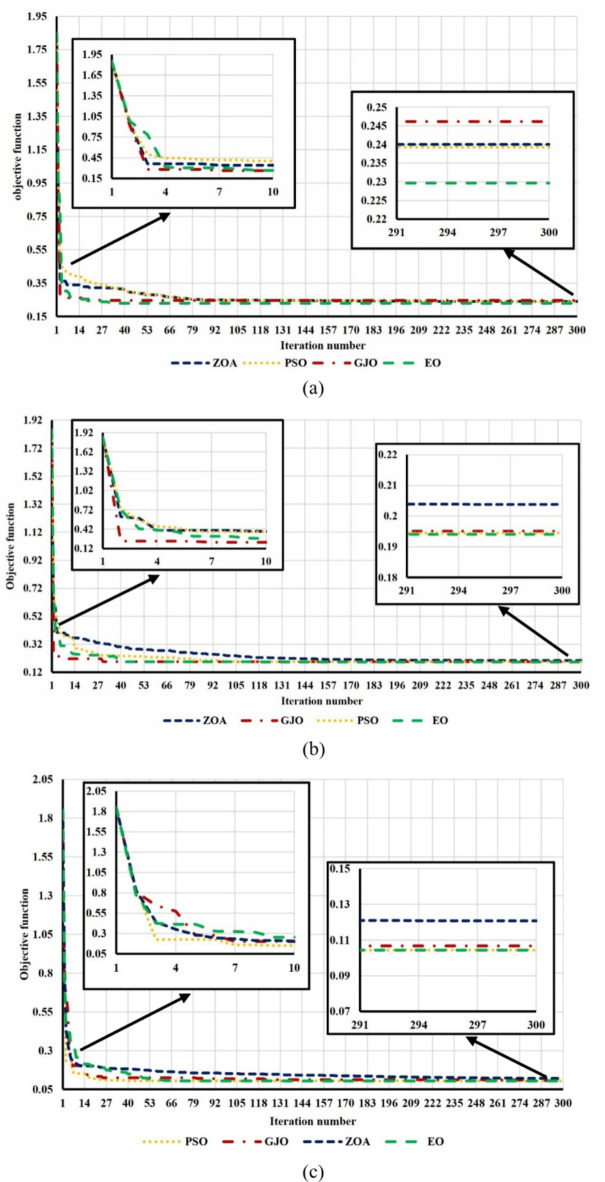
Convergence curves of ZOA, PSO, GJO, and EO for the IEEE 33 bus system at *h* = 13: (**a**) case II, (**b**) case III, and (**c**) case IV.

**Fig. 23:**
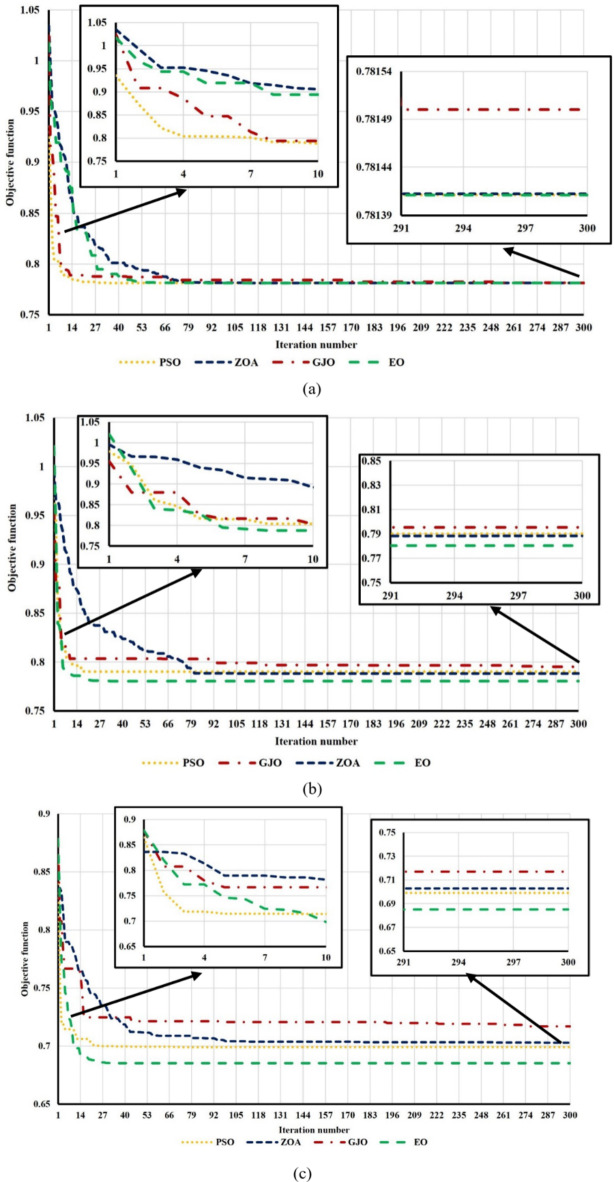
Convergence curves of ZOA, PSO, GJO, and EO for the Cairo 59-bus system at *h* = 15: (**a**) case II, (**b**) case III, and (**c**) case IV.

For both test systems, EO achieved the least objective function values in most cases compared to the other optimizers. In addition, EO showed the best robustness, as indicated by a comparison of its robustness factor with other optimizers. Furthermore, there was relatively slight variation between the best and worst values, indicating that all the runs nearly converged to the best solution. For the previous reasons, the EO algorithm is selected in this article. However, the time consumed for the complete run was not the best in all cases; EO was chosen because our planning problem was offline. Finally, the superiority of the EO performance compared to other optimizers is shown in Figs. 22 and 23.

**Table 4:** Comparison between the best and worst objective function values of the selected optimizers in solving cases II, III, and IV in the IEEE 33-bus test system at *h* = 13.

Case	Technique	Objective function						*T*_*complete*_ (s)	$\:{N}_{best}$	$\:\alpha\:\:$(%)
		Best			Worst					
		Value	EV demand (MW)	DG capacity (MW)	Value	EV demand (MW)	DG capacity (MW)			
Case II	ZOA	0.2400285	21.559	24.422	0.2514	18.7546	22.1867	342	6	60
	GJO	0.2461695	21.335	25.253	0.2585	17.2848	20.396	237	4	40
	PSO	0.2392966	21.275	25.2991	0.2505	20.5493	24.1544	110	7	70
	EO	0.2296940	21.55	25.296	0.2299	21.5134	25.2667	175	8	80
Case III	ZOA	0.2038330	31.974	34.9936	0.2052	30.0316	33.0608	333	7	70
	GJO	0.1951212	30.617	33.8827	0.2851	14.4763	18.6036	272	5	50
	PSO	0.1944894	30.172	33.412	0.1963	29.907	32.949	190	8	80
	EO	0.1940644	30.772	33.792	0.1945	30.772	33.771	260	8	80
Case IV	ZOA	0.1207623	35.261	37.845	0.1259	33.147	35.6288	547	4	40
	GJO	0.1066719	38.178	41.0319	0.1939	16.738	18.9767	489	3	30
	PSO	0.1043194	38.465	41.271	0.1518	21.871	25.221	605	5	50
	EO	0.1043194	38.465	41.271	0.1069	36.926	39.75	472	7	70

**Table 5:** Comparison between the best and worst objective function values of the selected optimizers in solving cases II, III, and IV in the Cairo 59-bus test system at *h* = 15.

Case	Technique	Objective function						*T*_*complete*_ (s)	$\:{N}_{best}$	$\:\alpha\:$ (%)
		Best			Worst					
		Value	EV demand (MW)	DG capacity (MW)	Value	EV demand (MW)	DG capacity (MW)			
Case II	ZOA	0.7814127	58.8	70.0976	0.7934	54.669	70.107	1560	4	40
	GJO	0.7819616	58.71	69.7	0.7823	58.645	69.548	1260	6	60
	PSO	0.7815113	58.8	70.11	0.7967	56.9999	68.872	780	5	50
	EO	0.7814	58.8	70.123	0.7821	57.9	70.116	1320	7	70
Case III	ZOA	0.7882706	65.855	75.1997	0.7883	65.84	75.4	1500	7	70
	GJO	0.7952946	65.175	75.76	0.7968	65.176	73.89	1407	6	60
	PSO	0.790124	66.855	75.2866	0.7914	65.408	74.55	1200	6	60
	EO	0.7804653	66.76	76.215	0.7879	65.74	75.65	1140	7	70
Case IV	ZOA	0.7029	83.488	81.447	0.7242	77.467	78.284	2130	4	40
	GJO	0.7170228	73.297	82.15	0.7978	69.13	70.32	1410	3	30
	PSO	0.6990108	82.8	82.202	0.7156	78.33	79.45	1135	5	50
	EO	0.6852	87.066	83.701	0.7074	80.477	81.146	1620	6	60

## Conclusions

### Problem addressed

It is believed that two of the keys to sustainable development are renewable distributed generation (RDG) and electric vehicles (EVs) that are integrated into distribution networks (DNs). Nevertheless, several technical issues might occur when integrating RDG and EV charging demands on a wide scale. Consequently, the distribution system operator (DSO) has two responsibilities: first, precisely define the combined distributed generation and electric vehicle hosting capacity, abbreviated DG-EV-HC, and second, attempt to support the DN by implementing efficient and cost-effective enhancement techniques to increase its ability to host additional RDG and EV demand without violating technical aspects.

### Relevance of the problem

The majority of publications in the available literature have focused on DG-HC assessment and enhancement without taking into account the impact of EV charging demand and vice versa. Additionally, there are extremely few publications that simultaneously optimize EV-HC and DG-HC. Furthermore, a number of strategies have been presented in the literature to improve DG-HC or EV-HC independently. Nevertheless, improving the combined DG-EV-HC requires an extensive analysis to determine and implement the best combination that can significantly support both DG-HC and EV-HC simultaneously while maintaining the grid’s safe operation. Thus, this problem poses a significant obstacle that needs to be overcome.

### Proposed solution

This paper presents a novel synergistic enhancement strategy to increase the combined DG-EV-HC by combining Volt/VAR control of SIs with a dynamic pricing-based DR. In order to implement the proposed approach, a hierarchical bi-level optimization framework is developed and solved by the equilibrium optimiser (EO), which outperformed other optimisers in terms of solving the suggested solution. Furthermore, in order to ensure the robustness of the suggested solution, three distinct load categories—residential, industrial, and commercial—as well as the charging types for electric vehicles at home and at public stations are taken into consideration. In addition to the IEEE 33-bus system, a real DN with 59 buses in Cairo, Egypt is used to verify the suggested solution’s scalability.

### Key findings and their implications

The results demonstrated, first, the critical importance of taking into account the charging demand for EVs through DG-HC. When EVs are integrated, the estimated DG-HC may increase by almost 133%. In addition, the positive role of applying DR programs to enhance the combined DG-EV-HC is obviously highlighted. Given that DR is one of the most cost-effective enhancement techniques, it can raise both DG-HC and EV-HC by almost 27% and 34%, respectively, in some cases, demonstrating its effectiveness. In addition, the importance of switching from applying single enhancement techniques to hybrid enhancement techniques has been discussed. When the proposed DR solution is supported with the SIs Volt/VAR control, a superior enhancement is achieved. Therefore, the proposed synergistic enhancement approach significantly improved the DG-HC and EV-HC by almost 49.2% and 61.2%, respectively.

Future work directions will be concentrated on considering uncertainty and variability as the current article could be expanded to consider the inherent uncertainty and variability related to renewable energy generation, electric vehicles charging demand, and other system parameters to produce more robust and adaptive HC enhancement approaches. Since the focus of this paper is on the technical aspects of HC augmentation, we will also take regulatory factors into account. Future work could also involve economic elements, such as the cost-benefit study of the proposed approaches, as well as the legal and policy frameworks that might encourage the extensive implementation of these techniques.

## Data Availability

The datasets used and/or analysed during the current study available from the corresponding author on reasonable request.
